# Kraft Lignin as a Partial Filler Replacement in Asphalt Mixtures with Nanosilica-Modified Bitumen: Volumetric, Mechanical, Moisture-Related and Rheological Properties

**DOI:** 10.3390/ma19204386

**Published:** 2026-10-19

**Authors:** Ömer Genç, Başak Varli Bingöl

**Affiliations:** Department of Civil Engineering, Çankırı Karatekin University, 18100 Çankırı, Türkiye; omergenc980@gmail.com

**Keywords:** kraft lignin, nanosilica, asphalt mixture, filler replacement, moisture susceptibility, indirect tensile strength, recovered binder rheology

## Abstract

Kraft lignin, an aromatic biopolymer from pulping, has mostly been studied as a binder modifier. Its use as a mineral-filler replacement has received little attention at the mixture level. This study evaluated the effect of kraft lignin replacing 25% and 75% of the limestone filler in a dense-graded asphalt mixture (dry process), alone and combined with 0.5% and 1.0% nanosilica blended into a 50/70 bitumen (wet process) as a modifier of the lignin effect. Volumetric properties, Marshall stability and flow, indirect tensile strength (ITS) before and after AASHTO T 283 conditioning, and recovered-binder rheology were measured. The materials were also examined by Fourier-transform infrared spectroscopy (FTIR) and scanning electron microscopy (SEM). Lignin lowered the voids in the mineral aggregate (VMA), effective binder content and air voids, and raised unconditioned ITS from 864 kPa to 1204–1232 kPa, but the tensile strength ratio (TSR) fell from 93.2% to 60.4% and 44.0%. Combined with nanosilica, the lignin mixtures gained a further 10–20% in unconditioned ITS, and at 75% replacement their TSR rose to 58.0–60.3%, a significant gain, though still below the control value. Recovered-binder rheology, determined once per binder, responded non-monotonically to nanosilica dosage. Lignin thus increased unconditioned strength at the expense of moisture resistance, and nanosilica offset only part of this loss.

## 1. Introduction

Asphalt pavements depend on bitumen, a petroleum-derived binder, and on large volumes of mineral aggregate and filler. Reducing the demand for virgin petroleum products and finding uses for industrial by-products have become central goals in pavement materials research. Many bio-based and functional additives are being evaluated for this purpose [1,2,3]. Waste tyre rubber, for example, has been used both in rubber-modified asphalt surface mixtures and as tyre-derived aggregate in the subgrade, and the rubber-modified mixtures showed higher cracking resistance than conventional mixtures [4]. Lignin, the largest natural source of aromatic structures, is among the most widely studied of these candidates [1,5]. Kraft pulping dominates world chemical pulp production. More than 70 million tonnes of kraft lignin could be extracted each year, but most of it is currently burned for energy at the pulp mill [6]. Only a small share of the lignin produced worldwide, estimated at 1–2%, is recovered for use in materials [1]. Kraft lignin is a highly branched polymer rich in phenolic and aliphatic hydroxyl groups [6]. These groups account for much of its appeal as an asphalt modifier, and also for some of the concerns about using it.

Most studies have added lignin to the binder, either as a modifier or as a partial bitumen extender [1,3,7]. Its phenolic groups can scavenge free radicals, and lignin-modified binders have formed fewer carbonyl and sulfoxide groups during ageing and shown better ageing indices [3,8,9]. Lignin also tends to stiffen the binder. In a study combining experiments with molecular dynamics simulation, both the rutting failure temperature and the bitumen–aggregate interaction energy rose with lignin content, while fatigue and self-healing performance declined [10]. In mixtures, adding 5% lignin by mass of binder increased Marshall stability by up to 21% and moisture resistance by up to 13%, at the cost of fatigue life [11]. At low dosages, on the other hand, kraft lignin has been found to act largely as an inert filler in the binder [12]. In aged bitumen modified with 15–20% lignin, the higher lignin content gave higher stiffness and a less regular particle distribution [13,14]. Lignin has also been combined with polymers and used in warm-mix binders [15,16].

The effect of lignin on moisture damage is less clear-cut. Depending on dosage, its polar groups can either reduce or aggravate water-induced damage [3]. Surface free energy measurements have been used to quantify the moisture susceptibility of lignin-modified binders with natural aggregates [17]. When lignin was added as a fibre instead of a powder, rutting and thermal-cracking resistance improved but freeze–thaw stability decreased [18]. Combining lignin fibre with other fibres has been proposed to balance these effects [19]. Replacing part of the mineral filler with lignin is a different case. The filler controls the stiffness of the mastic and its interaction with the binder [20,21], and organic additives in the mastic may act mainly through physical stabilisation rather than chemical modification of the binder [22]. Kraft lignin (1.2 g/cm^3^) is also far less dense than limestone filler (2.78 g/cm^3^), so an equal-mass replacement increases the volume of the filler fraction. Moisture damage stems from loss of adhesion at the binder–aggregate interface and loss of cohesion within the binder or mastic [23], and it is sensitive to the air-void content of the mixture [24]. A lignin-rich mastic could affect both mechanisms.

Nanosilica is one of the nanomaterials most often used to modify bitumen. Because of its large specific surface area, it raises the viscosity, softening point and rutting-related rheological parameters of the binder [25,26,27]. Nanosilica-modified mixtures have shown higher indirect tensile strength, Marshall stability and tensile strength ratio (TSR) than unmodified mixtures [28,29,30]. It has also been reported to improve bitumen–aggregate adhesion, especially with limestone aggregate [31]. The gains depend heavily on dosage and dispersion. Ageing resistance fell once the nanosilica content exceeded an optimum [32], and agglomeration at higher dosages, together with the absence of standard dispersion procedures, is a recurring problem [33,34,35]. Nanosilica has also been paired with polymers [36], crumb rubber [37] and waste polymers in warm-mix asphalt [38] to compensate for the drawbacks of the primary modifier. In the same way, nanosilica might compensate for some of the drawbacks of lignin. In the present study, it is therefore used as a secondary additive that modifies the lignin effect, not as the subject of the investigation.

Most studies have added lignin to the binder and assessed it mainly through binder rheology. Mixture-level studies are fewer and have mostly used lignin as a binder modifier, extender or fibre [1,3,7,9,11,18]. More recent mixture-level studies have likewise used lignin to replace 8.5–30% of the bitumen [39,40,41]. Reviews note that lignin added by the dry process acts mainly as a particulate filler [42]. However, its use as a deliberate replacement for the mineral filler, and the resulting change in filler volume, have received little attention at the mixture level, especially with respect to moisture susceptibility. The present study differs in three respects. First, kraft lignin is used as a partial replacement for the mineral filler at replacement levels of up to 75%, and its effect on moisture susceptibility is assessed at the mixture level. Second, the lignin effect is examined in combination with nanosilica to test whether nanosilica can modify it and, in particular, offset any loss of moisture resistance associated with lignin. To the authors’ knowledge, this combination has not been examined before. Third, the work extends the authors’ earlier studies of lignin in aged bitumen [13,14] to mixtures prepared with a virgin 50/70 binder.

Dense-graded asphalt mixtures were therefore prepared in which kraft lignin replaced 25% or 75% of the limestone filler, alone or with 0.5% or 1.0% nanosilica by mass of bitumen. The primary aim was to determine the effect of lignin, alone and in combination with nanosilica. The lignin-containing mixtures were therefore compared with the control mixture, and the hybrid mixtures were also compared with the corresponding lignin-only mixture, to show how nanosilica modified the lignin effect. Volumetric properties, Marshall stability and flow, indirect tensile strength before and after moisture conditioning, and the high-temperature rheology of the recovered binders were determined. Fourier-transform infrared (FTIR) spectroscopy and scanning electron microscopy (SEM) were used to characterise the materials and mixtures.

## 2. Materials and Methods

**Figure 1:**
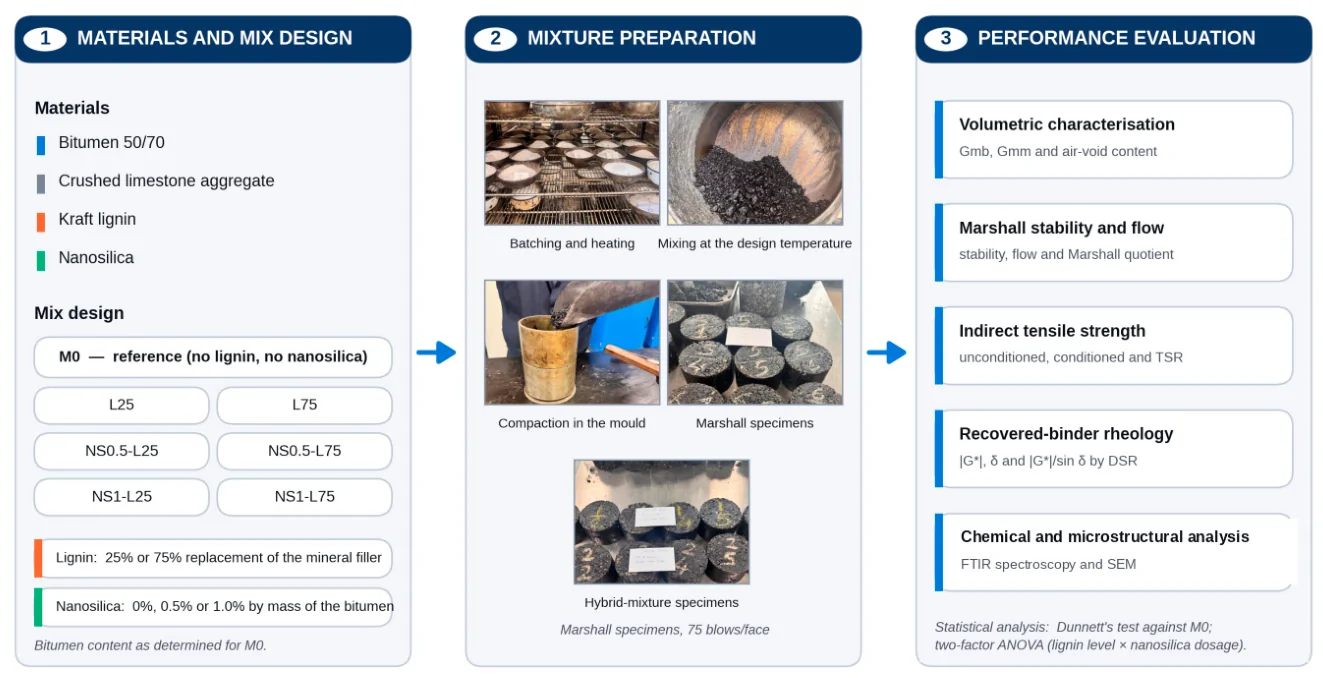
Flow chart of the experimental programme.

The experimental programme is summarised in Figure 1.

### 2.1. Materials

Crushed limestone coarse aggregate, fine aggregate and mineral filler were obtained from a single quarry in Ankara, Türkiye, and all fractions were supplied by the same producer. Table 1 gives the physical properties of the aggregate.

A 50/70 penetration-grade bitumen, widely used in Türkiye, was obtained from an asphalt plant in Ankara; its properties are listed in Table 2.

Alkali (kraft) lignin (Ataman Chemicals, Istanbul, Türkiye) was used to replace part of the mineral filler and was incorporated by the dry process (Section 2.2). Its properties, as reported by the producer, are given in Table 3.

Nanosilica (SiO_2_) was used as a bitumen modifier and incorporated by the wet process (Section 2.2); its physicochemical characteristics are also listed in Table 3.

**Figure 2:**
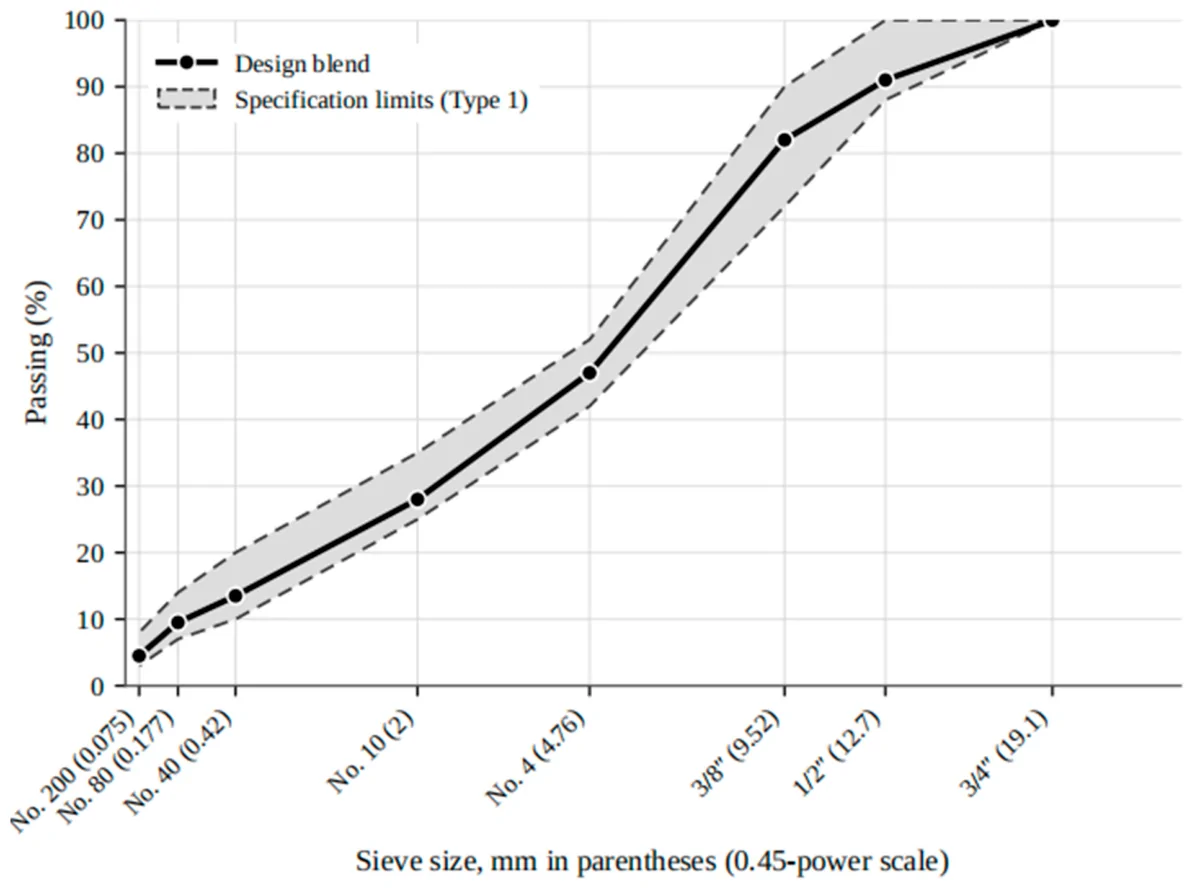
Design gradation of the aggregate blend plotted on a 0.45-power scale.

The aggregate blend was proportioned to the Type 1 wearing-course envelope of the Turkish General Directorate of Highways specification [50]. This corresponds to a dense-graded mixture with a nominal maximum aggregate size of 12.5 mm (maximum size 19.0 mm), which also meets the AASHTO M 323 [51] control points for that size. The design gradation is shown in Figure 2.

### 2.2. Mixture Design and Specimen Preparation

The optimum bitumen content of the control mixture (M0), determined by Marshall mix design, was 5.1% by mass of aggregate (4.85% by total mixture mass). This content was kept constant for all mixtures. It was not re-optimised for the modified mixtures, so that all mixtures were compared at the same binder content and compaction effort. The resulting differences in volumetric composition are quantified in Section 3.1.

Lignin was added by the dry process. It replaced part of the mineral filler passing the 0.075 mm sieve (4.5% by mass of the aggregate blend) on an equal-mass basis and was added to the heated aggregate before the binder. Kraft lignin (1.2 g/cm^3^) is less than half as dense as the limestone filler (apparent specific gravity 2.779). The equal-mass replacement therefore increased the solid volume of the filler fraction by approximately 33% at 25% replacement and by 99% at 75% replacement. The gradation of the fractions retained on the 0.075 mm sieve was the same for all seven mixtures. Nanosilica was added to the bitumen by the wet process before mixing with the aggregate. The bitumen was heated to 150–155 °C, nanosilica was added in a single step at 0.5% or 1.0% by mass of bitumen, and the blend was dispersed with a high-shear mixer at 1400 rpm for 60 min. The modified bitumen was mixed with the aggregate immediately after dispersion, without intermediate storage, to limit additional ageing of the binder. The dispersion of nanosilica in the binder was not verified directly, for example by microscopy of the modified binder or by a storage-stability test. Seven mixtures were prepared: the control M0; two lignin-only mixtures, L25 and L75; and four hybrid mixtures, NS0.5-L25, NS1-L25, NS0.5-L75 and NS1-L75. The number after L is the percentage of mineral filler replaced, and the number after NS is the nanosilica dosage as a percentage by mass of bitumen. Their compositions are given in Table 4. Mixtures containing nanosilica without lignin were not included, because the programme was designed around lignin: nanosilica was included only as a modifier of the lignin effect, not as an additive in its own right. The nanosilica effects reported here therefore describe how nanosilica modified the lignin-containing mixtures and cannot be separated into an independent effect of nanosilica and a lignin–nanosilica interaction.

Specimens were compacted with 75 blows per face in accordance with EN 12697-30 [52]. Mixing and compaction were carried out at 150 °C, and the mixture was reheated to the compaction temperature after being transferred to the moulds. At the optimum binder content, M0 had approximately 4% air voids and met the wearing-course design criteria.

### 2.3. Methods

#### 2.3.1. Volumetric Characterisation

Bulk specific gravity (Gmb) was measured by the saturated surface-dry method (EN 12697-6 [53], procedure B; ASTM D2726 [54]) and maximum theoretical specific gravity (Gmm) by the pycnometer method (EN 12697-5 [55]; AASHTO T 209 [56]). Air-void content (Va) was calculated according to EN 12697-8 [57]. Voids in the mineral aggregate (VMA), voids filled with asphalt (VFA), absorbed binder content (Pba) and effective binder content (Pbe) were calculated from Gmb and Gmm with the volumetric relationships given in AASHTO R 35 [58]. In these calculations, lignin was treated as part of the solid phase, with the density reported by the producer (1.2 g/cm^3^). The apparent specific gravity was used for the limestone filler because its bulk specific gravity was not determined, so the resulting values are approximate.

#### 2.3.2. Marshall Test

Marshall stability and flow were measured according to EN 12697-34 [59] and ASTM D6927 [60]. The Marshall quotient (MQ, kN/mm) was calculated as stability divided by flow, using mean values for M0 and individual measurements for the modified mixtures.

Three specimens were tested for M0 and one for each modified mixture, so the Marshall results were evaluated descriptively.

#### 2.3.3. Indirect Tensile Strength and Moisture Conditioning

Indirect tensile strength (ITS, kPa) was measured according to EN 12697-23 [61] at 25 ± 0.5 °C and a loading rate of 50 mm/min.

Unconditioned and conditioned subsets were chosen to have similar mean air-void contents. Moisture conditioning followed AASHTO T 283 [62], including a freeze–thaw cycle, with two modifications. First, the Marshall-compacted specimens had the air-void contents reported in Table 5 rather than 7.0 ± 0.5%. Second, each subset contained six specimens for M0 and two for each modified mixture. All specimens were compacted with the same effort as in the mix design (75 blows per face), so that the mixtures were compared at a common compaction effort rather than at a common air-void content. Conditioned specimens were vacuum-saturated to the 70–80% degree of saturation specified in AASHTO T 283. TSR was calculated as the ratio of conditioned to unconditioned mean ITS, expressed as a percentage.

Statistical comparisons were based on individual ITS measurements (Section 2.4).

#### 2.3.4. Rheology of the Recovered Binder

Binder was extracted with trichloroethylene (ASTM D2172 [63]; EN 12697-1 [64]) and recovered with a rotary evaporator (EN 12697-3 [65]). Dynamic shear rheometer measurements followed AASHTO T 315 [66] at 10 rad/s from 58 to 94 °C in 6 °C steps. A comparative index temperature was obtained by logarithmic interpolation at |G*|/sin δ = 2.2 kPa, where |G*| is the complex shear modulus and δ the phase angle. This value is the threshold for short-term-aged binder in AASHTO M 320 [67]; no performance grade was assigned. Since |G*|/sin δ has known limitations as an indicator of rutting resistance [68], the index was used only to compare the recovered binders with one another. Each recovered binder was tested once. The programme covered the six lignin-containing mixtures, with L25 and L75 serving as the respective references.

#### 2.3.5. FTIR Spectroscopy and Scanning Electron Microscopy

Fourier-transform infrared (FTIR) spectra were recorded over 4000–400 cm^−1^ on a Bruker Tensor II spectrometer (Bruker Optik GmbH, Ettlingen, Germany) fitted with a diamond attenuated total reflectance accessory (Platinum ATR) and a DLaTGS detector, at 4 cm^−1^ resolution with 24 sample scans and 32 background scans (Blackman–Harris three-term apodisation, zero-filling factor 2). The neat 50/70 bitumen used in the mixtures, the kraft lignin and the nanosilica were measured as received. The six lignin-containing mixtures (L25, NS0.5-L25, NS1-L25, L75, NS0.5-L75 and NS1-L75) were measured on fragments taken from fractured Marshall specimens; M0 was not measured. The neat bitumen was measured in a separate session on the same instrument and accessory. Spectra were exported without smoothing, baseline correction or atmospheric compensation. In ATR measurements, band intensities depend on the contact between the sample and the crystal [69,70], and the mixture fragments contained mineral filler as well as binder. The spectra were therefore compared by band position rather than absolute intensity, using characteristic band positions of lignin, silica, bitumen and calcium carbonate from the literature [25,71,72,73,74,75]. The acquisition parameters and the laboratory sample codes are listed in Tables S1 and S2.

The microstructure of the same six mixtures was examined on fragments of fractured Marshall specimens using a field-emission scanning electron microscope (ZEISS Sigma 300 VP; Carl Zeiss Microscopy GmbH, Oberkochen, Germany), a technique widely applied to asphalt binders and mixtures [76]. The fragments were sputter-coated with gold and imaged under high vacuum at 20 kV with a secondary-electron (SE2) detector, at working distances of 4.6–9.2 mm. Two areas of each fragment were imaged at magnifications from 100× to 20,000×; the images compared in Section 3.5 were taken at 1000× and 5000× in the same area of each fragment. Energy-dispersive X-ray spectroscopy was not carried out. The imaging conditions and the complete image series are given in Table S4 and Figures S3–S8.

### 2.4. Statistical Analysis

How each property was analysed depended on the number of replicates available. ITS was analysed inferentially, with two specimens per mixture and conditioning state (six for M0), and air-void contents were compared with M0 (six specimens per modified mixture and twelve for M0). Marshall stability and flow (one specimen per modified mixture) and recovered-binder rheology (one determination per binder) are reported descriptively, without inferential tests. The inferential analyses addressed three questions: (i) whether nanosilica modified the effect of lignin on ITS, relative to the corresponding lignin-only mixture; (ii) how the lignin replacement level and nanosilica changed the retained strength (TSR); and (iii) how each lignin-containing mixture, with or without nanosilica, compared with M0, and whether the nanosilica effect on ITS persisted after accounting for specimen air-void content.

The analysis centred on the effect of lignin, alone and in combination with nanosilica. The effect of lignin relative to the control was tested by comparing each lignin-containing mixture with M0 (question iii). Its modification by nanosilica was tested within the six lignin-containing mixtures, which formed the primary analysis set. They formed a 2 × 3 factorial design, with two lignin replacement levels (25% and 75% of the mineral filler), three nanosilica dosages (0%, 0.5% and 1.0% by mass of bitumen) and two specimens per cell in each conditioning state. The control mixture M0 served as an external benchmark and was not included in the factorial models. Including a cell without either additive would have confounded the comparison between the two lignin replacement levels with the comparison between mixtures with and without lignin.

(i) Modification of the lignin effect on ITS by nanosilica: Four contrasts were specified in advance and evaluated separately for each conditioning state, each comparing a hybrid mixture with its lignin-only reference, L25 or L75. They were tested with two-sided *t* tests. The error term was the residual mean square of the full model with the lignin replacement level, nanosilica dosage and their interaction (six residual degrees of freedom), and the Holm correction [77] was applied across the four contrasts within each state. Differences, percentage changes and 95% confidence intervals (CIs) were calculated from unrounded means. Whether the nanosilica effect depended on the lignin replacement level was assessed through the lignin replacement level × nanosilica dosage interaction. Effect sizes are reported as partial eta squared (ηp^2^) [78].

(ii) Effects of lignin and nanosilica on retained strength: The TSR is a ratio of two means and has no replicate-based estimate of variability. The effect of the additives on retained strength was therefore evaluated on the natural logarithm of the individual strengths of the six lignin-containing mixtures. A three-factor model with the lignin replacement level, nanosilica dosage and conditioning state was used (24 observations; twelve residual degrees of freedom). In this model, an interaction between an additive factor and conditioning state corresponds to a difference in the tensile strength ratio. The additional effect of nanosilica on retained strength was expressed as the TSR: the TSR of each hybrid mixture divided by that of its lignin-only reference. On the logarithmic scale, this ratio is a contrast of four cell means. It was tested against unity with the Holm correction and is reported with a back-transformed 95% confidence interval. Extending the same formulation to all seven mixtures gave the comparison of each mixture with M0.

(iii) Air-void covariate and comparisons with M0: The association between nanosilica dosage and ITS was further explored by analysis of covariance (ANCOVA) of the individual specimens of the six lignin-containing mixtures. Specimen air-void content was the covariate, and the lignin replacement level and nanosilica dosage were the factors; the ANCOVA models included their main effects without an interaction term. Because the additives themselves affect air-void content, this analysis cannot separate their direct effects from the accompanying volumetric changes. Each modified mixture was compared with M0 for air-void content and ITS using Dunnett’s test [79]. A significance level of 0.05 was used throughout, and the original analyses were performed in Minitab 17 (Minitab LLC, State College, PA, USA). During revision, the affected volumetric calculations, Dunnett comparisons and ANCOVA results were independently checked using Python 3.12.14 with NumPy 2.3.5 and SciPy 1.17.0.

## 3. Results and Discussion

ITS is reported as mean ± one standard deviation. The results are presented from the perspective of the lignin effect. The lignin-only mixtures are compared first with M0 to show the effect of lignin alone. The hybrid mixtures are then compared with their lignin-only reference, L25 or L75, to show how nanosilica modified this effect, and with M0 to show the combined effect. The reference for each percentage change is stated explicitly.

### 3.1. Volumetric Properties

Table 4 gives the composition and volumetric properties of the seven mixtures. Replacing mineral filler with lignin lowered the air-void content from 4.33% for M0 to 3.37% for L25 and 3.59% for L75, and both differ significantly from M0 (*p* ≤ 0.002). Nanosilica reduced it further. In the L25 series, air voids fell steadily from 3.37% to 3.11% and 2.89% as the nanosilica dosage increased. In the L75 series, 0.5% nanosilica had only a marginal effect, whereas 1.0% nanosilica lowered it to 3.28%. Lignin thus changes the volumetric composition relative to the control, and nanosilica was accompanied by a further drop in air voids, most clearly at the 1% dosage. These lower air-void contents need to be kept in mind when interpreting the Marshall and ITS results, because changes in mechanical behaviour cannot be attributed solely to the direct action of the additives.

The volumetric calculation (Section 2.3.1) indicates why the air-void content fell although the binder content was unchanged. Because the equal-mass replacement placed a larger volume of solids in the filler fraction, VMA decreased from approximately 14.8% for M0 to 13.4% for L25 and 12.8% for L75 (12.7–13.3% for the hybrid mixtures). The calculated absorbed binder rose from about 0.4% for M0 to 0.6% for L25 and 0.9% for L75. As a result, the effective binder content fell from 4.5% to 4.3% and 4.0% by mass of mixture, and the volume ratio of filler to effective binder increased from about 0.35 to 0.50 and 0.79. VFA ranged from 71% to 78% for all mixtures. The lignin-containing mixtures thus had a filler-rich mastic with less effective binder than the control. Their VMA was also below the minimum of 14.0% specified in AASHTO M 323 for a 12.5 mm nominal maximum aggregate size. These values are approximate: they depend on the assumed specific gravities of the filler and lignin, and the higher calculated binder absorption may partly reflect uncertainty in the density of lignin rather than actual absorption.

### 3.2. Marshall Stability, Flow and Marshall Quotient

**Figure 3:**
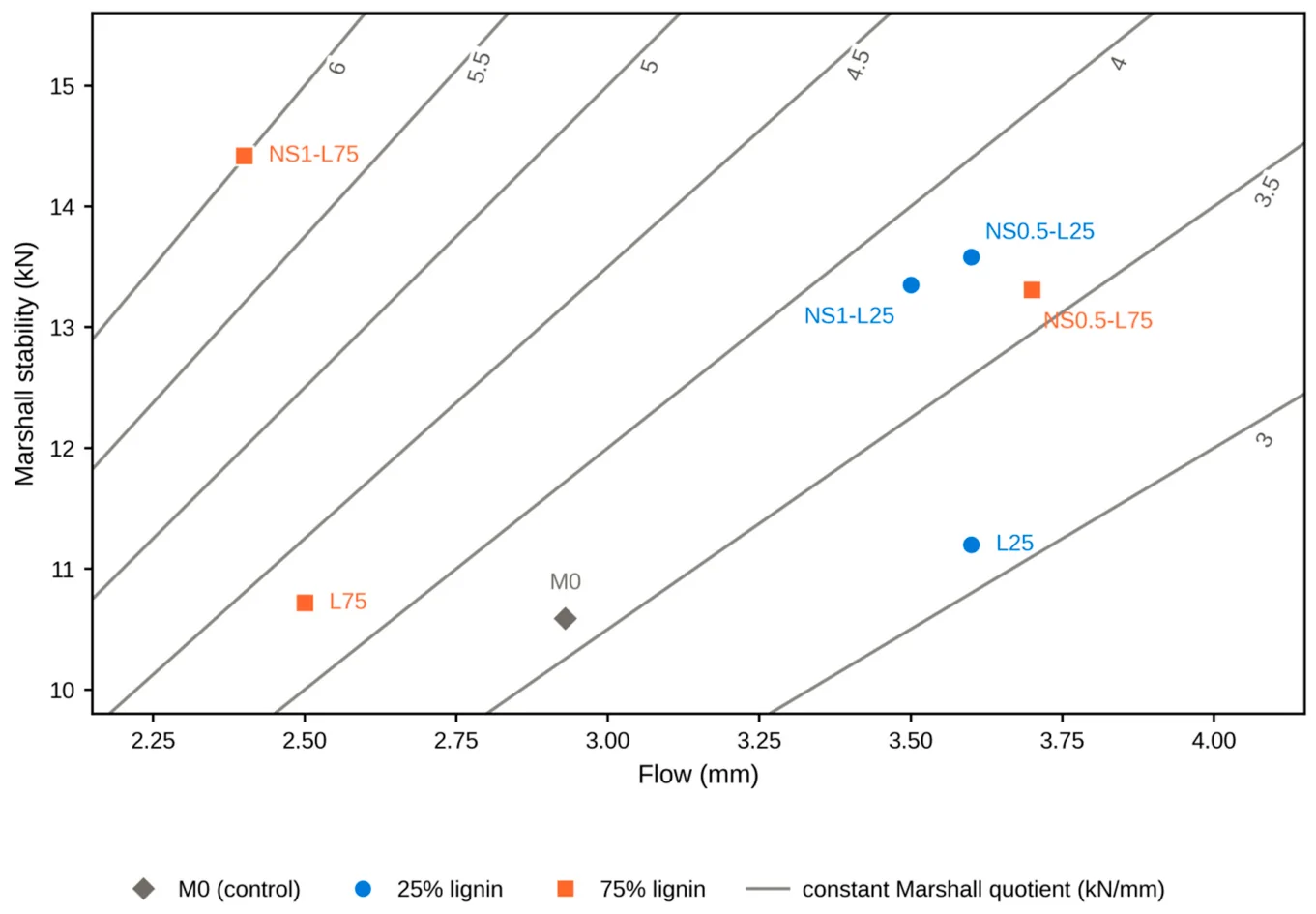
Marshall stability and flow: mean values for M0 (*n* = 3) and individual measurements for the modified mixtures (*n* = 1). Grey lines indicate constant stability-to-flow ratios (Marshall quotient, MQ).

Figure 3 presents the measured Marshall stability and flow, with MQ represented by lines of constant ratio. The changes associated with lignin replacement are considered first, relative to M0, followed by the additional effect of nanosilica relative to L25 or L75. All comparisons in this section are descriptive, since replicate specimens were available only for the control mixture. For M0, the coefficients of variation of the three specimens were 4.8% for stability and 7.9% for flow. The differences in stability between the lignin-only mixtures and M0 (+1.2% and +5.7%) are of the same order and cannot be distinguished from specimen-to-specimen scatter, whereas the increases associated with nanosilica (19–35%) are clearly larger. Flow, and therefore MQ, is the less certain of the two measurements.

Lignin replacement alone changed the measured Marshall stability only slightly: 11.20 kN for L25 and 10.72 kN for L75, compared with 10.59 kN for M0 (+5.7% and +1.2%). Flow differed more clearly, rising from 2.93 mm for M0 to 3.60 mm for L25 but falling to 2.50 mm for L75, so that MQ was 3.61, 3.11 and 4.29 kN/mm, respectively. With nanosilica, measured stability rose to 13.58 and 13.35 kN in the L25 series (+21.3% and +19.3% relative to L25) and to 13.31 and 14.42 kN in the L75 series (+24.2% and +34.5% relative to L75). The flow response depended on dosage. In the L25 series MQ rose to 3.77 and 3.82 kN/mm, whereas in the L75 series it fell from 4.29 to 3.60 kN/mm at 0.5% nanosilica (−16.1%) and rose to 6.01 kN/mm at 1.0% (+40.1%).

Nanosilica thus increased the measured Marshall stability of the lignin-containing mixtures at both dosages, while its effect on MQ depended on how flow responded. NS1-L75 had the highest measured stability and MQ, whereas NS0.5-L75 shows that higher stability can go together with a lower MQ than that of the lignin-only reference. Increases in Marshall stability have also been reported for nanosilica-modified [29] and lignin-modified mixtures [11].

### 3.3. Indirect Tensile Strength and Moisture Susceptibility

**Figure 4:**
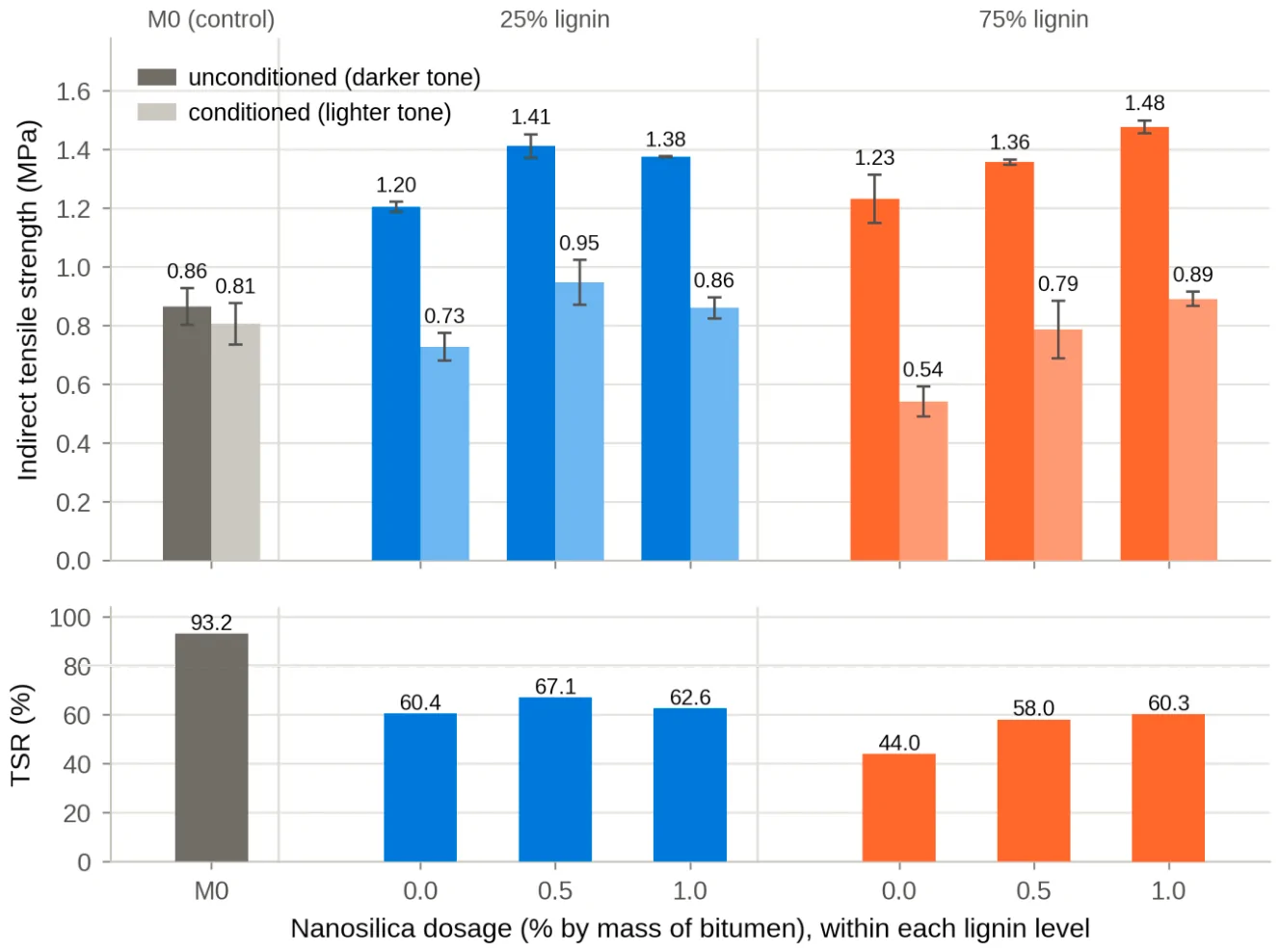
Unconditioned and conditioned indirect tensile strength and tensile strength ratio of the investigated mixtures. Bars show mean values and error bars ±1 standard deviation. Grey, blue and orange denote M0 and the 25% and 75% lignin series, respectively; darker and lighter tones denote the unconditioned and conditioned states, respectively.

Lignin replacement raised mean unconditioned ITS from 864 kPa for M0 to 1204 kPa for L25 and 1232 kPa for L75 (Figure 4). After conditioning, the corresponding strengths were 806, 728 and 542 kPa, and TSR dropped from 93.2% to 60.4% and 44.0%, respectively. The gain in unconditioned strength came at the cost of poorer strength retention, especially at 75% lignin replacement.

**Figure 5:**
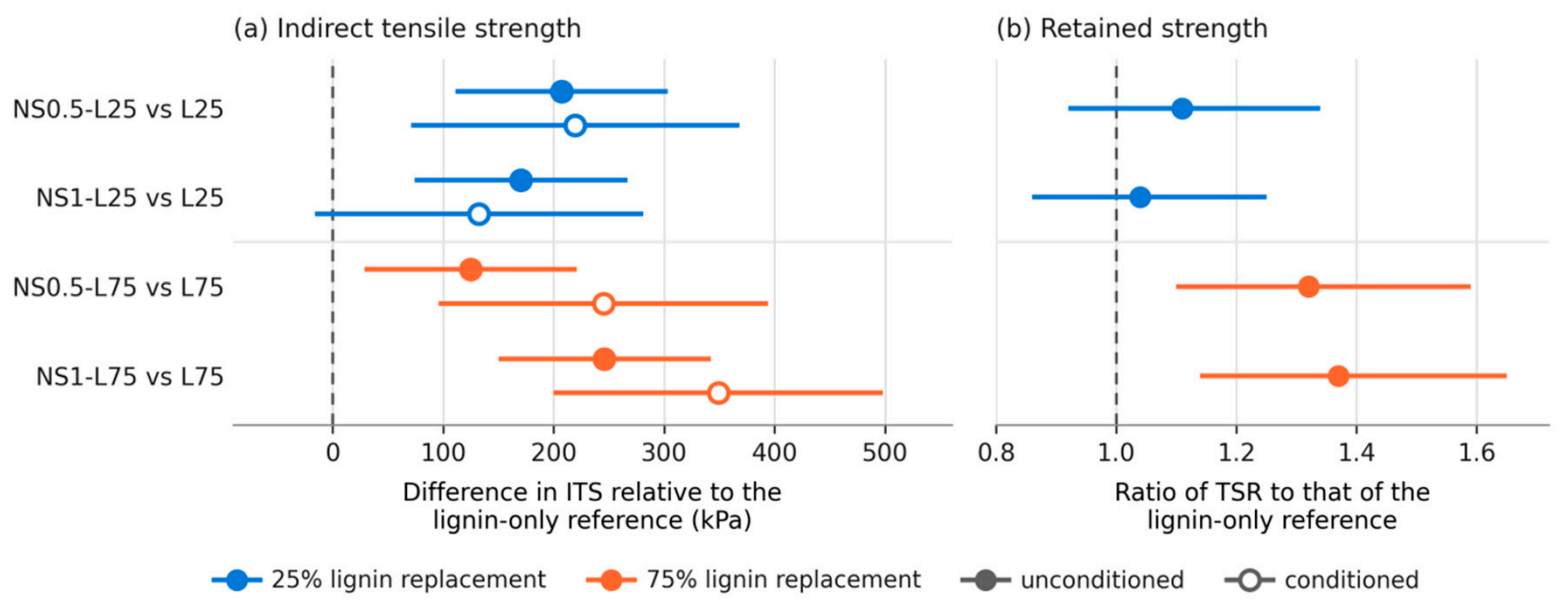
Modification of the lignin effect by nanosilica, relative to the corresponding lignin-only mixture: (**a**) difference in indirect tensile strength in the unconditioned and conditioned states, and (**b**) tensile strength ratio (TSR) relative to the reference. Points are estimates and bars are 95% confidence intervals; a value of zero in (**a**) and of unity in (**b**) denotes no effect. Differences were calculated from unrounded means (*n* = 2 per group and state) using the residual mean square of the full factorial model (df = 6); the ratios in (**b**) use the three-factor model on the logarithmic scale (df = 12). Holm-adjusted *p*-values are given in the text.

All four hybrid mixtures exceeded their lignin-only references in unconditioned ITS, by 125–246 kPa (10.2–20.0%; Figure 5a); every 95% confidence interval excluded zero, and Holm-adjusted *p*-values ranged from 0.003 to 0.019. Conditioned ITS increased by 18.2–30.1% relative to L25 and by 45.2–64.4% relative to L75. Three of these contrasts were significant; the exception was NS1-L25 (+132 kPa, 95% CI −16 to +281 kPa, adjusted *p* = 0.072).

In a two-factor analysis of the six lignin-containing mixtures, unconditioned ITS was associated with nanosilica dosage (*F*(2,6) = 31.41, *p* < 0.001, ηp^2^ = 0.913) but not with the lignin replacement level (*F*(1,6) = 1.20, *p* = 0.315). Conditioned ITS was associated with both the lignin replacement level (*F*(1,6) = 9.02, *p* = 0.024) and nanosilica dosage (*F*(2,6) = 20.23, *p* = 0.002). The lignin replacement level × nanosilica dosage interaction was not significant when the two conditioning states were analysed separately (*p* = 0.078 and *p* = 0.086). It was significant, however, when they were analysed together on the logarithmic scale (*F*(2,12) = 6.01, *p* = 0.016, ηp^2^ = 0.500).

The unconditioned and conditioned subsets of each mixture had closely matched mean air-void contents, differing by at most 0.34 percentage points. The degree of saturation of all conditioned specimens of the modified mixtures was within the 70–80% target range (70.8–78.3%). Two of the six conditioned M0 specimens fell marginally below it; excluding them would lower the TSR of M0 from 93.2% to 90.0%, still well above that of any modified mixture. However, differences in air-void content between mixtures may also have contributed to the observed moisture-related response. One possible explanation is the hydrophilic character of kraft lignin, whose hydroxyl groups could promote water uptake at the binder–filler interface [3,6]; this mechanism was not examined here and remains a hypothesis. The FTIR and SEM results (Section 3.5) give no indication of a new phase or of a chemical change in the binder, which is consistent with a physical mechanism of this kind.

The volumetric changes described in Section 3.1 offer a second explanation, which may act together with the first. A larger volume of filler relative to effective binder may stiffen the mastic and reduce the thickness of the binder films around the aggregate. This would be expected to raise the tensile strength of unconditioned specimens but to make the mixture more vulnerable to loss of cohesion and adhesion once water has entered. The high unconditioned ITS of L75 (1232 kPa) together with its low TSR (44.0%) is consistent with this interpretation, which was not tested directly. A higher unconditioned tensile strength is not, by itself, evidence of better durability.

In the three-factor log-ITS model, both additives interacted with conditioning. The lignin replacement level × conditioning interaction was significant (*F*(1,12) = 11.66, *p* = 0.005, ηp^2^ = 0.493), reflecting the lower retained strength at 75% replacement. The nanosilica dosage × conditioning interaction was also significant (*F*(2,12) = 6.16, *p* = 0.014, ηp^2^ = 0.507), reflecting the higher retained strength of the nanosilica-containing mixtures. The three-way interaction was not significant (*F*(2,12) = 2.77, *p* = 0.102).

At 75% lignin replacement, nanosilica raised TSR from 44.0% to 58.0% at 0.5% dosage and to 60.3% at 1.0% dosage (Figure 5b). The corresponding TSRs relative to L75 were 1.32 (95% CI 1.10–1.59; adjusted *p* = 0.021) and 1.37 (1.14–1.65; adjusted *p* = 0.012). At 25% replacement the ratios were 1.11 (0.92–1.34) and 1.04 (0.86–1.25), neither significantly different from unity. The improvement was therefore statistically demonstrable only in the L75 series, although the non-significant three-way interaction does not establish that the two lignin levels responded differently.

Relative to M0, lignin lowered TSR by 32.8 and 49.2 percentage points at 25% and 75% replacement, respectively. Nanosilica recovered 6–20% of this loss in the L25 series and 28–33% in the L75 series. Improvements in moisture resistance with nanosilica have been attributed to better bitumen–aggregate adhesion [30,31]. Adhesion was not measured in this study.

**Figure 6:**
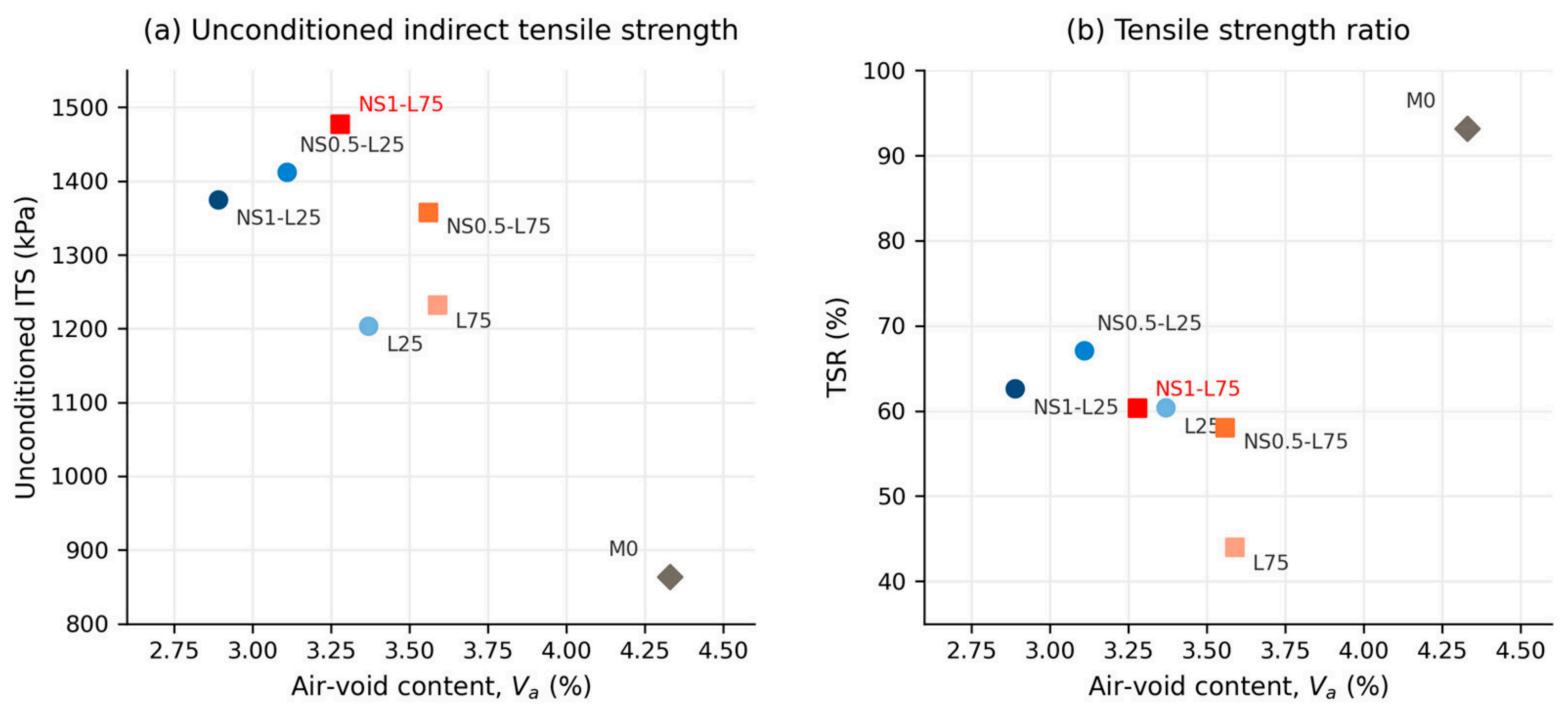
(**a**) Unconditioned indirect tensile strength and (**b**) tensile strength ratio against air-void content. Each point is a mixture mean; air-void contents are those reported in Table 4. These plots are descriptive; the covariance analyses used specimen-level indirect tensile strength and air-void content separately for the unconditioned and conditioned states.

An exploratory ANCOVA examined ITS in the six lignin-containing mixtures with specimen air-void content as the covariate; Figure 6 shows the corresponding relationships at the mixture level. The covariate was not significant in either conditioning state (unconditioned: *F*(1,7) = 4.06, *p* = 0.084; conditioned: *F*(1,7) = 0.04, *p* = 0.841), whereas the nanosilica term remained significant in both (*F*(2,7) = 19.41, *p* = 0.001 and *F*(2,7) = 9.08, *p* = 0.011, respectively).

Compared with M0, all modified mixtures had higher unconditioned ITS (Dunnett-adjusted *p* < 0.001). After conditioning, only L75 differed significantly, by −264 kPa (*p* = 0.002; Table 5).

The TSRs relative to M0 were 0.65 (95% CI 0.55–0.76) for L25, 0.47 (0.40–0.56) for L75 and 0.62–0.72 for the hybrid mixtures (all Holm-adjusted *p* < 0.001). Nanosilica thus reduced, but did not eliminate, the loss of strength retention caused by lignin; no lignin-containing mixture reached the TSR of the control. Higher absolute ITS should not be mistaken for recovery of moisture resistance.

### 3.4. Rheological Behaviour of the Recovered Binder

**Figure 7:**
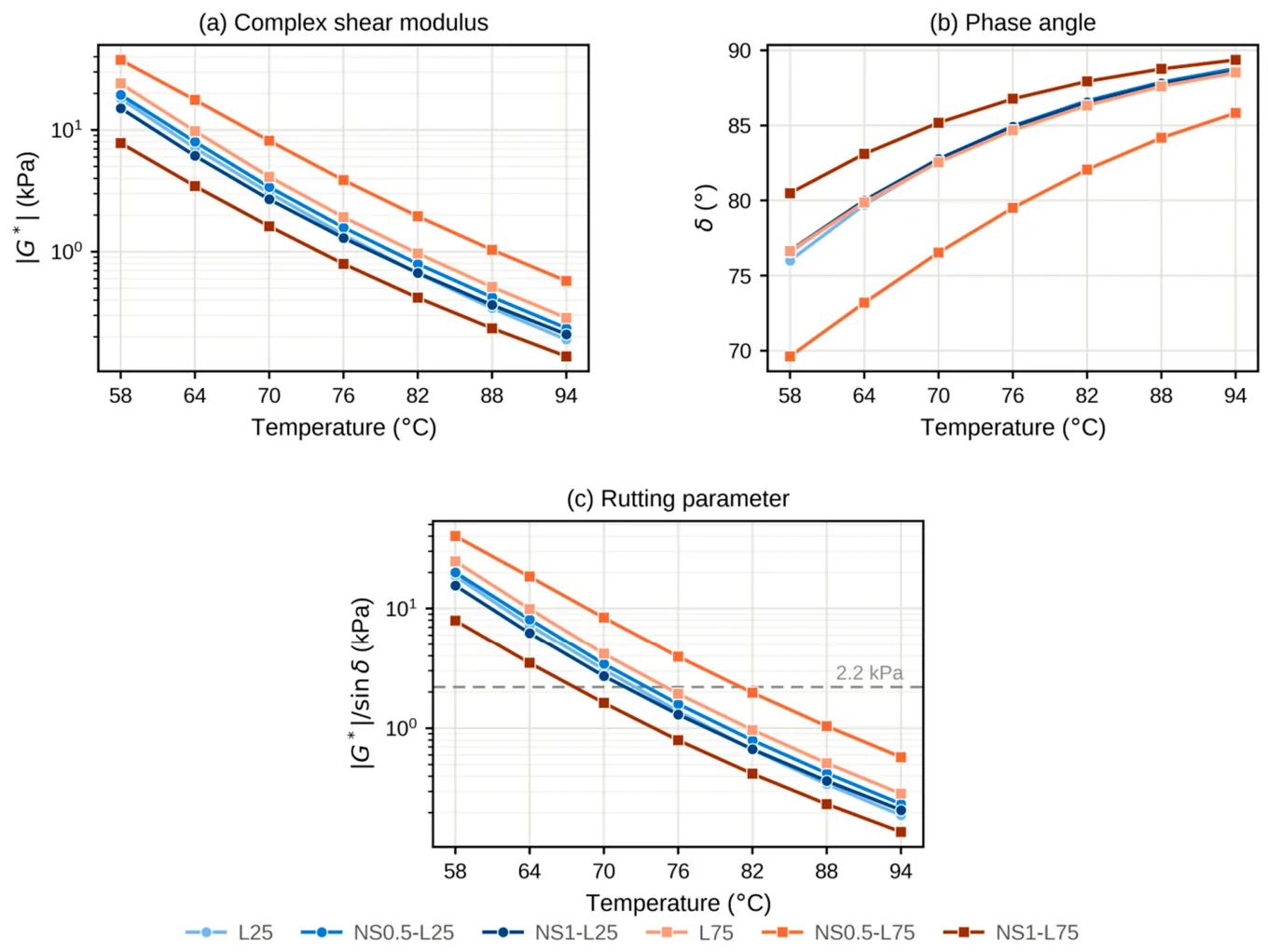
Temperature-dependent rheological response of the recovered binders: (**a**) complex shear modulus, (**b**) phase angle, (**c**) rutting parameter. The dashed line in (**c**) marks the 2.2 kPa threshold used to determine the comparative index temperature.

**Figure 8:**
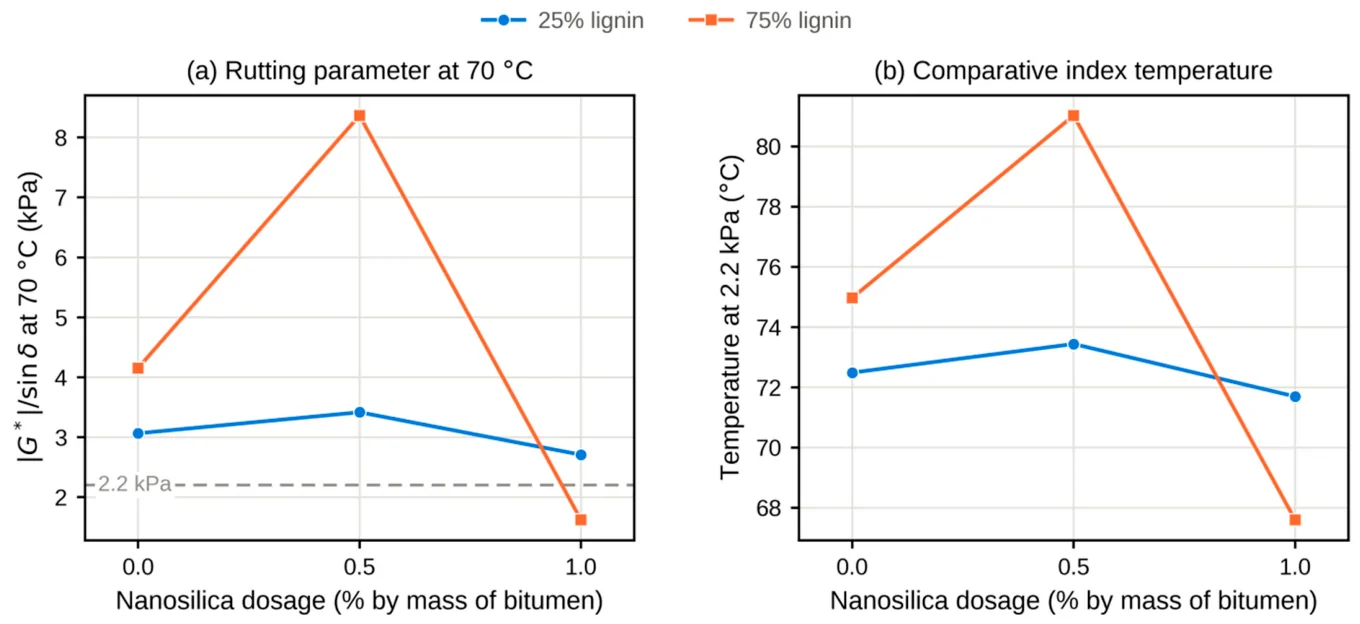
Variation in the rheological behaviour with nanosilica dosage at the two levels of lignin replacement: (**a**) rutting parameter at 70 °C and (**b**) comparative index temperature. The dashed line in (**a**) marks the 2.2 kPa threshold.

Figure 7 and Figure 8 show the rheological response of the binders recovered from the lignin-containing mixtures. Because no binder was recovered from M0, these comparisons show how nanosilica modified the response of the lignin-containing binders relative to the lignin-only reference, L25 or L75; the effect of lignin itself on binder rheology cannot be assessed from these data. For all recovered binders, |G*| and |G*|/sin δ decreased and the phase angle increased with temperature (Figure 7).

For the L25 reference, the rutting parameter at 70 °C was approximately 3.1 kPa and the comparative index temperature 72.5 °C. Adding 0.5% nanosilica raised these to approximately 3.4 kPa and 73.4 °C, whereas 1.0% nanosilica lowered them to approximately 2.7 kPa and 71.7 °C. The lower dosage thus produced a modest increase in high-temperature stiffness, and increasing the dosage brought no further benefit.

The dose response was more pronounced in the L75 series. Compared with approximately 4.2 kPa and 75.0 °C for L75, NS0.5-L75 reached approximately 8.4 kPa and 81.0 °C. It also had the highest complex shear modulus and the lowest phase angle of all the recovered binders. NS1-L75, however, gave approximately 1.6 kPa at 70 °C and an index temperature of 67.6 °C, both below the L75 values. In short, the rheological response did not improve consistently with nanosilica dosage. A loss of benefit above an optimum nanoparticle dosage has been reported before and is usually attributed to agglomeration and incomplete dispersion [32,33,35]. The present measurements do not identify its cause, since the dispersion of nanosilica in the binder was not verified. The non-parallel trends in Figure 8 suggest that the two lignin replacement levels responded differently to nanosilica, but they do not establish a statistically significant interaction.

The rheological and moisture-related responses followed different dosage trends. In the L75 series, both nanosilica dosages raised TSR relative to L75, whereas the comparative index temperature rose at 0.5% nanosilica and fell at 1.0%. Higher retained tensile strength thus did not consistently coincide with a higher rheological index of the recovered binder. The two measurements capture different aspects of behaviour: strength retention after moisture conditioning at the mixture level, and high-temperature rheology of the recovered binder. In addition, the rheological comparisons rest on single determinations per binder (Section 3.6).

### 3.5. Chemical and Microstructural Characterisation

**Figure 9:**
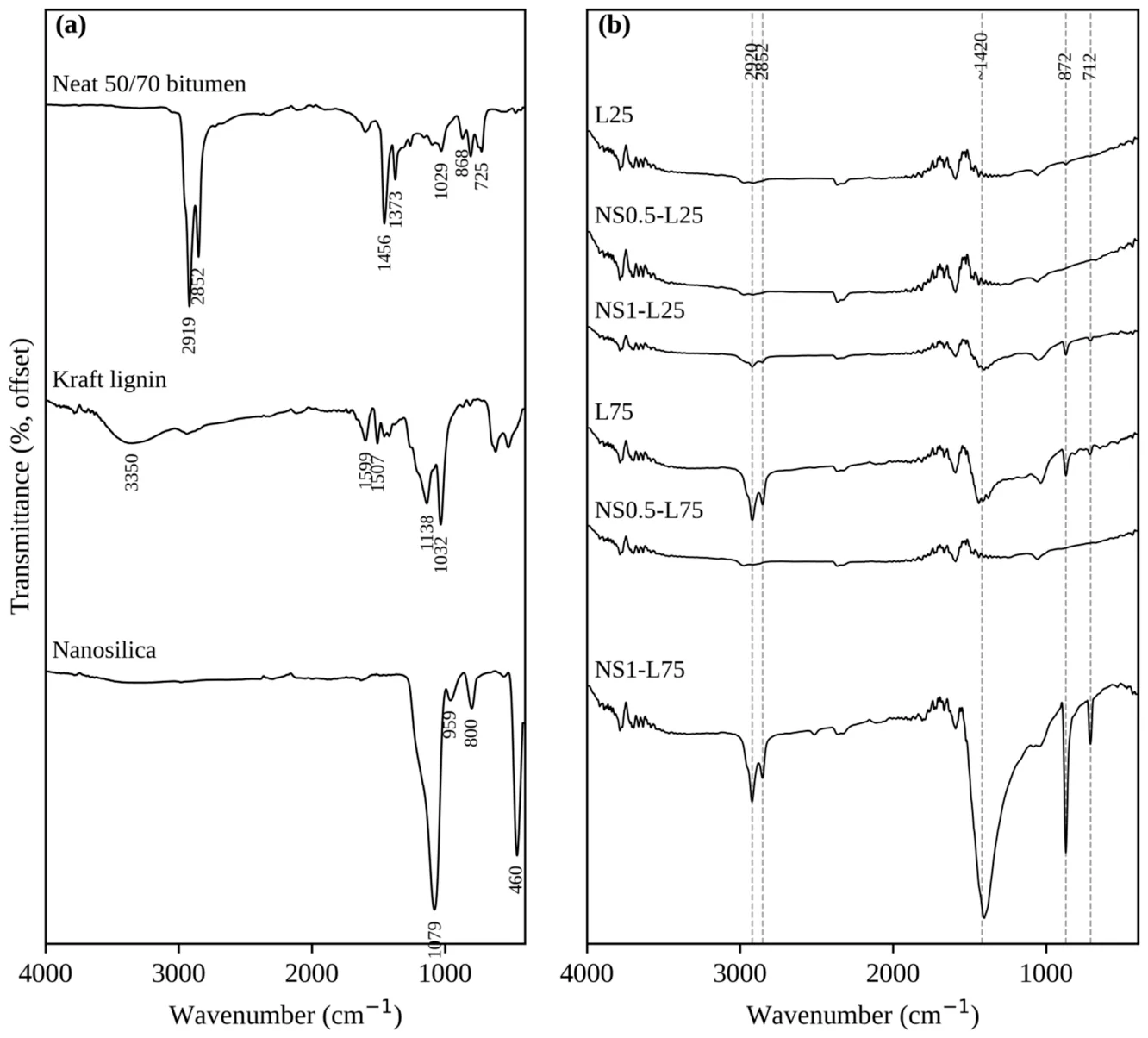
Fourier-transform infrared (FTIR) spectra of (**a**) the neat 50/70 bitumen, kraft lignin and nanosilica and (**b**) fragments of the six lignin-containing mixtures. Spectra are shown as measured and vertically offset for clarity; dashed lines in (**b**) mark the aliphatic C–H bands of the binder (2920 and 2852 cm^−1^) and the carbonate bands of the limestone filler (~1420, 872 and 712 cm^−1^).

Figure 9a shows the spectra of the neat bitumen and the two additives. The neat 50/70 bitumen gave the aliphatic C–H stretching bands at 2919 and 2852 cm^−1^, the CH_2_ and CH_3_ bending bands at 1456 and 1373 cm^−1^, the sulfoxide S=O band at 1029 cm^−1^ and the aromatic C–H out-of-plane bending bands at 868 and 808 cm^−1^ [71,72]; the kraft lignin gave a broad O–H stretching band centred at 3350 cm^−1^ and aromatic skeletal vibrations at 1599 and 1507 cm^−1^ [73]; and the nanosilica gave the Si–O–Si bands at 1079, 800 and 460 cm^−1^ together with the Si–OH band at 959 cm^−1^ [25,32]. The spectra of the mixture fragments (Figure 9b) contained only the aliphatic C–H bands of the binder and the calcium carbonate bands of the limestone filler at approximately 1420, 872 and 712 cm^−1^ [74,75], in addition to atmospheric water-vapour and carbon-dioxide bands. These signals were weak and varied between fragments with the local proportion of binder and filler in contact with the crystal, so they were not used to compare the mixtures, and the Si–O–Si band of nanosilica was not resolved at dosages of at most 1.0% by mass of binder. The individual spectra and the observed band positions are given in Figures S1 and S2 and Table S3.

**Figure 10:**
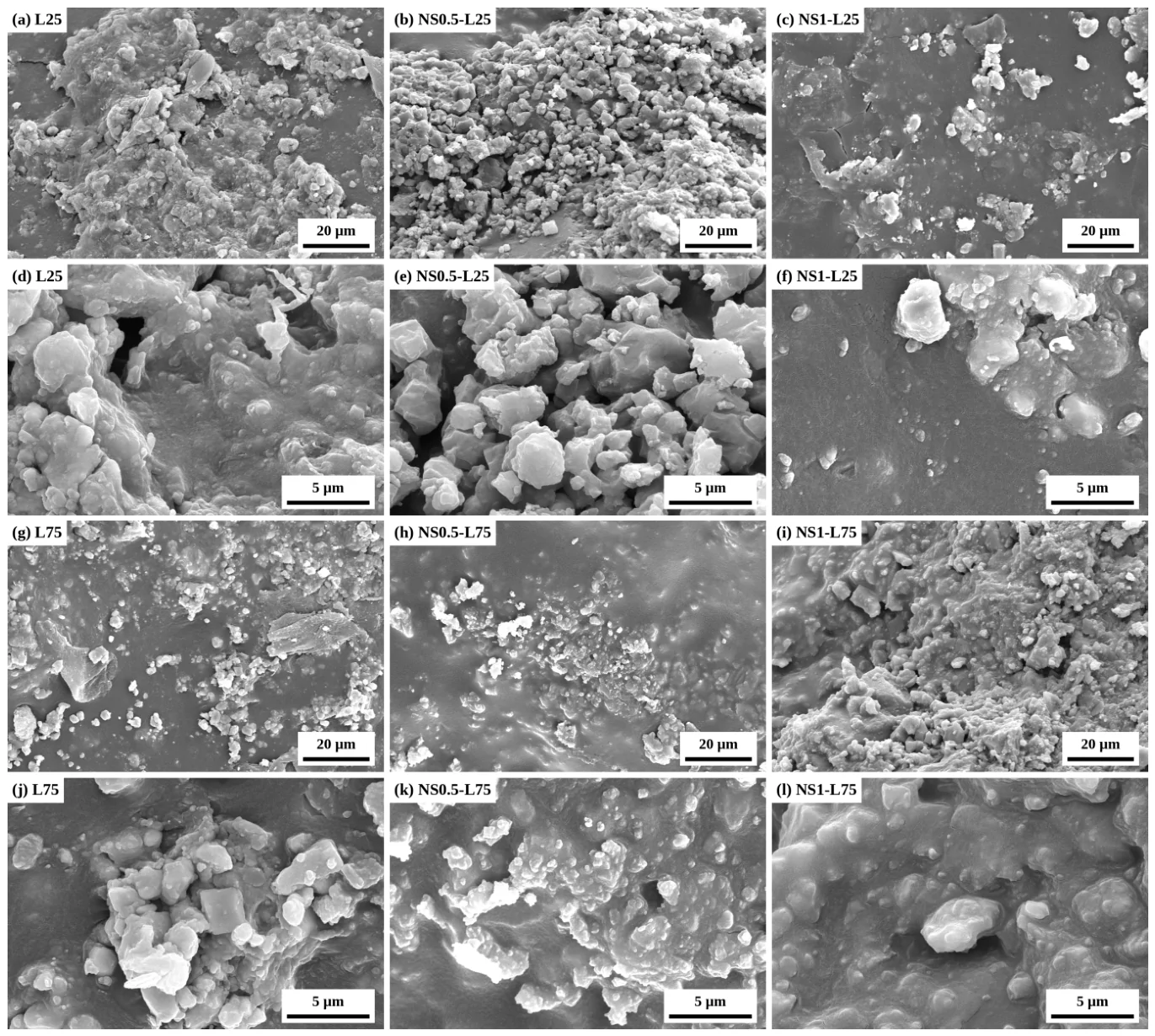
Scanning electron microscopy (SEM) images of fracture surfaces of the lignin-containing mixtures: (**a**–**c**) L25 series at 1000×, (**d**–**f**) L25 series at 5000×, (**g**–**i**) L75 series at 1000× and (**j**–**l**) L75 series at 5000×. Columns show 0%, 0.5% and 1.0% nanosilica. Images at 1000× and 5000× were taken in the same area of each fragment (secondary-electron (SE2) detector, 20 kV, gold-coated).

Figure 10 compares the fracture surfaces of the six lignin-containing mixtures at 1000× and 5000×. In every mixture the surface consisted of a continuous binder phase with embedded particles, mostly a few micrometres in size, which at 5000× were coated with binder and had rounded edges; gaps at the particle–binder boundary were rare. The proportion of particles differed between fields of view, but, with one fragment per mixture and without elemental analysis, these differences cannot be assigned to the additives, and the primary nanosilica particles (15–35 nm) could not be resolved at these magnifications. The complete image series is given in Figures S3–S8. The FTIR and SEM results therefore show no new phase and no band other than those of the binder and the limestone filler, which points to the larger filler volume and the altered mastic (Section 3.1 and Section 3.3), rather than to a chemical modification of the binder, as the origin of the observed mechanical and moisture-related changes.

### 3.6. Limitations and Scope

The experimental programme was designed as a screening study of the effect of lignin as a filler replacement, alone and in combination with nanosilica, and the level of replication reflects this aim. ITS was determined on two specimens per mixture and conditioning state, with six specimens per state for M0. Marshall stability and flow were measured on three specimens for M0 and one specimen for each modified mixture. Recovered-binder rheology was determined once for each of the six lignin-containing mixtures. With two specimens per cell, the homogeneity of variance assumed by the pooled residual term could not be checked formally, so the confidence intervals should be regarded as approximate. The fact that the nanosilica effect had the same direction in all four hybrid formulations nevertheless supports the main ITS findings. The Marshall and rheological results are presented as descriptive trends.

Because no binder was recovered from M0, the rheological comparisons describe the effect of nanosilica within the lignin-containing binders, not the effect of lignin relative to the neat binder. Solvent extraction and recovery may also alter the rheology of the recovered binder [80].

At constant binder content, the modified mixtures had lower air-void contents than M0, so comparisons with M0 reflect both the additives and the associated volumetric changes. Within the lignin-containing mixtures, the effect of nanosilica on ITS remained significant when specimen air-void content was included as a covariate, although this analysis cannot fully separate the two contributions.

The binder content was optimised for M0 only. Lignin replacement lowered VMA and the effective binder content (Section 3.1), but the modified mixtures were not evaluated at their own optimum binder contents. A design re-optimised for each mixture could lead to different volumetric and moisture-related results. The programme also did not include mixtures containing nanosilica without lignin, so the reported nanosilica effects apply only to lignin-containing mixtures, and the dispersion of nanosilica in the binder was not verified directly.

Finally, moisture susceptibility was evaluated at the air-void contents of the Marshall-compacted specimens (Table 5) rather than at the 7.0 ± 0.5% specified in AASHTO T 283. Since air-void content is known to influence moisture-damage susceptibility [24], the TSR values are used here to compare the mixtures with one another, not to assess them against specification limits. The modified mixtures had lower air-void contents than M0, while all their conditioned specimens reached the target saturation range (Section 3.3). Testing at a common air-void content would be needed to distinguish the contribution of volumetric differences from that of mixture composition to moisture susceptibility. Replicate testing, together with nanosilica-only mixtures, mixtures designed at their own optimum binder content and specimens at the standard air-void level, would allow these trends to be confirmed and generalised.

The FTIR and SEM examinations used single fragments of the six lignin-containing mixtures, did not include M0 and were not supported by elemental analysis. They document band positions and surface morphology, but they cannot identify the individual additive phases or show whether the additives interacted chemically with the binder.

## 4. Conclusions

This study examined the effect of replacing mineral filler with kraft lignin (dry process), alone and in combination with nanosilica added to the bitumen (wet process), on the volumetric, mechanical and moisture-related properties of asphalt mixtures and the high-temperature rheology of the recovered binder. Within the scope of the experimental programme, the main findings are as follows:

1. Replacing 25% and 75% of the mineral filler with kraft lignin on an equal-mass basis enlarged the filler volume and lowered VMA and the effective binder content. The air-void content fell from 4.33% (M0) to 3.37% (L25) and 3.59% (L75). The mechanical and moisture-related results therefore reflect both the additives and this change in volumetric composition.
2. Lignin raised unconditioned ITS from 864 kPa to 1204–1232 kPa but lowered TSR from 93.2% to 60.4% and 44.0%; higher unconditioned strength did not indicate better moisture resistance.
3. Nanosilica modified the lignin effect. Relative to the lignin-only mixtures, it increased unconditioned ITS by 10–20% in all four hybrid formulations and conditioned ITS in three of the four (Holm-adjusted *p* < 0.05). It reduced the loss of retained strength significantly only at 75% replacement, where TSR rose from 44.0% to 58.0–60.3%; no lignin-containing mixture reached the TSR of M0.
4. Marshall stability and flow (one specimen per modified mixture) and recovered-binder rheology (one determination per binder) were evaluated descriptively. In the lignin-containing mixtures, nanosilica was associated with higher Marshall stability, and the comparative index temperature of the recovered binder rose at 0.5% nanosilica but fell at 1.0%.
5. These findings were obtained at a binder content optimised for the control mixture, at air-void contents below the AASHTO T 283 level, with limited replication and without nanosilica-only mixtures. They should be confirmed under broader conditions before practical recommendations are made.

Overall, replacing the mineral filler with kraft lignin increased the unconditioned strength of the mixtures but reduced their moisture resistance, and nanosilica offset only part of this loss. Moisture resistance is therefore the property that limits the use of lignin as a filler replacement. Further work should concentrate on improving the moisture resistance of lignin-containing mixtures and on confirming these trends with additional independent replicates.

## Figures and Tables

**Table 1:** Physical properties of the aggregate and filler.

Property	Coarse Aggregate	Fine Aggregate	Filler	Standard
Bulk specific gravity	2.691	2.695	—	EN 1097-6 [43]/ASTM C127 [44]
Apparent specific gravity	2.740	2.752	2.779	EN 1097-6/ASTM C127
Water absorption (%)	0.663	0.766	—	EN 1097-6/ASTM C127

**Table 2:** Physical properties of bitumen.

Property	Unit	Value	Test Standard
Penetration at 25 °C	0.1 mm	52.4	ASTM D5 [45]
Softening point	°C	52	ASTM D36 [46]
Rotational viscosity at 135 °C	mPa·s	300	ASTM D4402 [47]
Specific gravity	—	1.037	ASTM D70 [48]
Flash point	°C	305	ASTM D92 [49]

**Table 3:** Properties of the additives, as provided by the producers.

Property	Unit	Kraft Lignin	Nanosilica (SiO_2_)
Purity	%	—	99.5
Particle size	nm	—	15–35
Passing 100 mesh	%	98.7	—
Passing 200 mesh	%	97.1	—
Specific surface area	m^2^/g	—	150–550
Density	g/cm^3^	1.2	2.2
Moisture content	% by mass	1	—
Ash content	% by mass	14	—
Structure	—	—	Porous
Colour	—	—	White

**Table 4:** Composition and basic volumetric properties of the investigated mixtures.

Mixture	Lignin (%)	NS (%)	Gmb	Gmm	Va (%)	ΔVa (pp)	*p* vs. M0
M0	0	0.0	2.414	2.523	4.33	—	—
L25	25	0.0	2.421	2.505	3.37	−0.97	<0.001
NS0.5-L25	25	0.5	2.422	2.500	3.11	−1.23	<0.001
NS1-L25	25	1.0	2.423	2.495	2.89	−1.44	<0.001
L75	75	0.0	2.370	2.458	3.59	−0.74	0.002
NS0.5-L75	75	0.5	2.368	2.455	3.56	−0.77	0.001
NS1-L75	75	1.0	2.370	2.450	3.28	−1.05	<0.001

Note: NS: nanosilica dosage (% by mass of bitumen); Gmb: bulk specific gravity; Gmm: maximum theoretical specific gravity; Va: air-void content; pp: percentage points. *p* vs. M0: adjusted *p*-value from Dunnett’s test. Differences were calculated from unrounded means. Va values are the means of six specimens for each modified mixture and of twelve specimens for M0; the corresponding standard deviations were 0.19–0.61 and 0.32 percentage points. The values reported in Table 5 are the means of the two-specimen subsets tested in each conditioning state and therefore differ slightly from those given here.

**Table 5:** Moisture-susceptibility data of the investigated mixtures and comparison with the control mixture M0.

Mixture	Unconditioned ITS (kPa)	Va, U (%)	Conditioned ITS (kPa)	Va, C (%)	S (%)	TSR (%)	ΔITS vs. M0, C (kPa)
M0	864 ± 63	4.33	806 ± 71	4.34	68.8–78.2 ^a^	93.2	—
L25	1204 ± 18	3.33	728 ± 47	3.67	70.8; 71.9	60.4	−78 (0.597)
NS0.5-L25	1412 ± 41	3.13	947 ± 77	3.15	72.9; 74.9	67.1	+141 (0.108)
NS1-L25	1375 ± 2	2.93	860 ± 36	2.97	76.6; 77.7	62.6	+54 (0.860)
L75	1232 ± 82	3.26	542 ± 51	3.29	71.7; 74.7	44.0	−264 (0.002)
NS0.5-L75	1357 ± 9	3.66	787 ± 98	3.68	73.1; 78.3	58.0	−19 (0.999)
NS1-L75	1477 ± 22	2.81	891 ± 25	3.08	71.7; 73.6	60.3	+85 (0.515)

Note: ITS: indirect tensile strength; TSR: tensile strength ratio. ITS values are mean ± one standard deviation (*n* = 2 for the modified mixtures; *n* = 6 for M0). Va: mean air-void content of the specimens in each subset; S: degree of saturation of the individual conditioned specimens; TSR calculated from unrounded means. ^a^ Range of the six conditioned M0 specimens. ΔITS vs. M0 is the difference from the control mixture in the conditioned state, with the Dunnett-adjusted *p*-value in parentheses; in the unconditioned state all six modified mixtures exceeded M0 by 340 to 613 kPa (*p* < 0.001 in every case).

## Data Availability

The raw data supporting the conclusions of this article will be made available by the authors on request.

## Supplementary Materials

The following supporting information can be downloaded at: https://www.mdpi.com/article/10.3390/ma19204386/s1, Table S1: Sample codes used in the FTIR and SEM raw data files; Table S2: FTIR acquisition parameters; Figure S1: Individual FTIR spectra of the neat bitumen, the additives and the six lignin-containing mixtures; Figure S2: FTIR spectra of the mixture fragments in the 1850–600 cm^−1^ region; Table S3: Band positions observed in the FTIR spectra; Table S4: SEM imaging conditions; Figures S3–S8: Complete SEM image series of mixtures L25, NS0.5-L25, NS1-L25, L75, NS0.5-L75 and NS1-L75.
