# Supplementary material for: Kraft Lignin as a Partial Filler Replacement in Asphalt Mixtures with Nanosilica-Modified Bitumen: Volumetric, Mechanical, Moisture-Related and Rheological Properties
Source: Materials (Basel). 2026 Oct 19;19(20):4386. doi: 10.3390/ma19204386 (PMC13706953; doi:10.3390/ma19204386)
Supplement: Supplementary file 1 [file materials-19-04386-s001.zip › materials-4607144-supplementary.pdf]

# Supplementary Materials

## Kraft Lignin as a Partial Filler Replacement in Asphalt Mixtures with Nanosilica-Modified Bitumen: Volumetric, Mechanical, Moisture-Related and Rheological Properties

**Ömer Genç and Başak Varli Bingöl ***

*Department of Civil Engineering, Çankırı Karatekin University, 18100 Çankırı, Türkiye*

* Correspondence: basakvarlibingol@karatekin.edu.tr

## Contents

Table S1. Sample codes used in the FTIR and SEM raw data files.
Table S2. FTIR acquisition parameters.
Figure S1. Individual FTIR spectra of the neat bitumen, the additives and the six lignin-containing mixtures.
Figure S2. FTIR spectra of the mixture fragments in the 1850–600 $cm^{-1}$ region.
Table S3. Band positions observed in the FTIR spectra.
Table S4. SEM imaging conditions.
Figures S3–S8. Complete SEM image series of the six lignin-containing mixtures.

## Section S1. Sample coding

The FTIR and SEM measurements were carried out by the Central Research Laboratory of Çankırı Karatekin University (ÇANKAM), which labelled the samples with its own codes. Table S1 links these codes to the mixture designations used in the article.

**Table S1.** Sample codes used in the FTIR and SEM raw data files.

| Mixture | Lignin (% of filler) | Nanosilica (% of bitumen) | SEM sample no. (image files) | FTIR file |
|---|---|---|---|---|
| L25 | 25 | 0 | 4 (4-047 to 4-062) | SDoruk filler lignin 4 |
| NS0.5-L25 | 25 | 0.5 | 3 (3-031 to 3-046) | SDoruk Lignin NS 3 |
| NS1-L25 | 25 | 1.0 | 6 (6-079 to 6-094) | SDoruk Lignin NS 6 |
| L75 | 75 | 0 | 1 (1-000 to 1-015) | SDoruk filler lignin 1 |
| NS0.5-L75 | 75 | 0.5 | 5 (5-063 to 5-078) | SDoruk Lignin NS 5 |
| NS1-L75 | 75 | 1.0 | 2 (2-016 to 2-030) | SDoruk Lignin NS 2 |
| Kraft lignin | — | — | — | SDoruk sarınumune |
| Nanosilica | — | — | — | SDoruk SrO2 [a] |
| Neat 50/70 bitumen | — | — | — | 50-70 [b] |

[a] Laboratory label; the spectrum is that of $SiO_2$ (Figure S1). [b] Measured on 6 August 2026 in a separate session with the same instrument; all other spectra were recorded on 11 September 2026. SEM images were recorded on 10 September 2026. M0 was not examined by FTIR or SEM.

## Section S2. FTIR spectroscopy

**Table S2.** FTIR acquisition parameters.

| Parameter | Value |
|---|---|
| Spectrometer | Bruker Tensor II (Bruker Optik GmbH, Ettlingen, Germany) |
| Accessory | Platinum ATR (A225/Q), single-reflection diamond crystal |
| Detector | RT-DLaTGS |
| Spectral range | 4000–400 $cm^{-1}$ |
| Resolution | 4 $cm^{-1}$ |
| Number of scans | 24 (sample); 32 (background) |

| | |
|---|---|
| Apodisation | Blackman–Harris three-term |
| Zero-filling factor | 2 (data point spacing ≈ 1.4 cm$^{-1}$) |
| Data processing | None (no smoothing, baseline correction or atmospheric compensation) |
| Samples | Powders (lignin, nanosilica) and bitumen as received; mixtures as fragments of fractured Marshall specimens |

Parameters were read from the OPUS (.0) files of the measurements and were identical for all nine spectra.

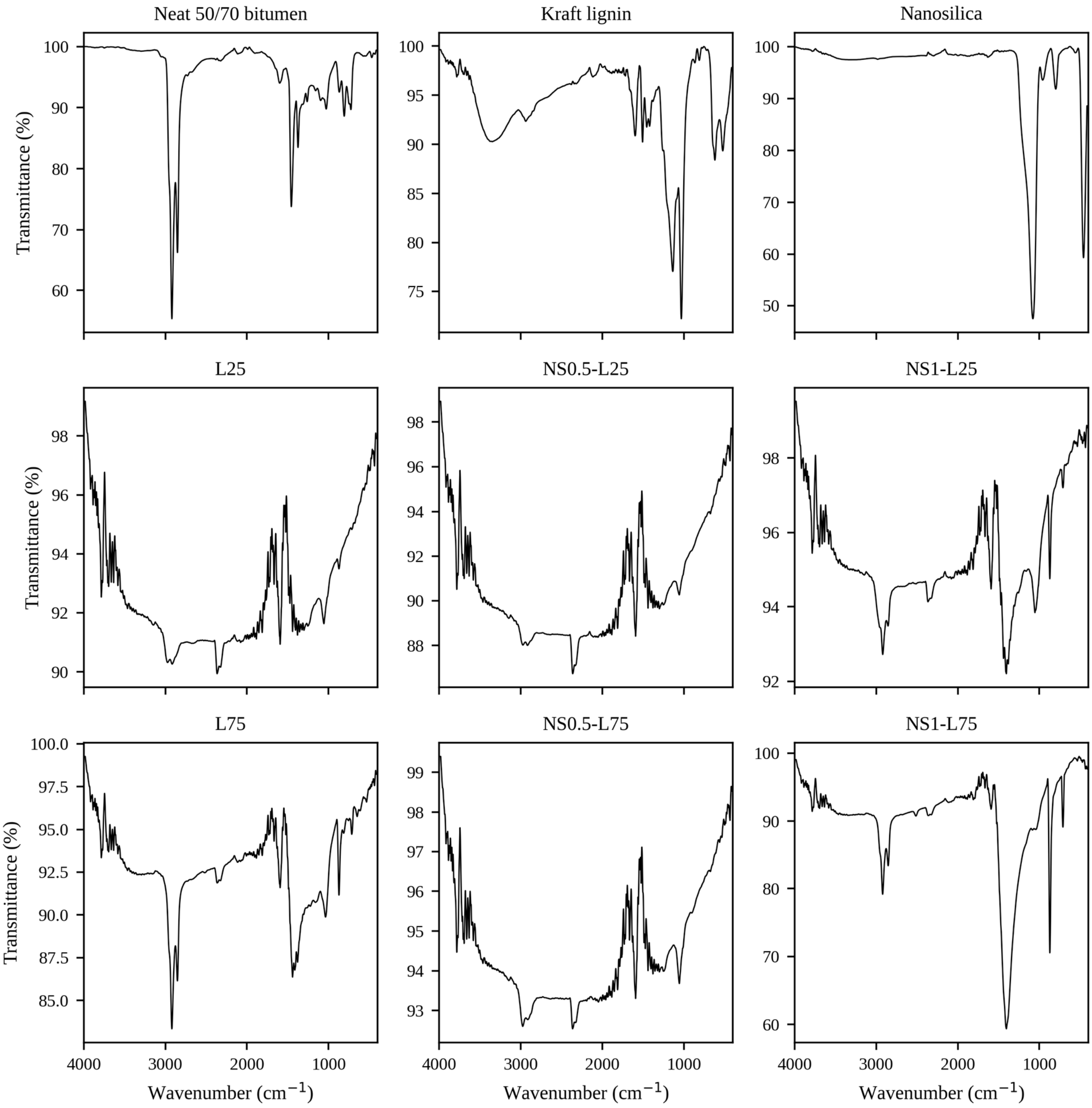


**Figure S1.** Individual FTIR spectra (as measured) of the neat 50/70 bitumen, kraft lignin, nanosilica and fragments of the six lignin-containing mixtures. Note the different transmittance scales. The mixture spectra show sharp atmospheric water-vapour bands (3900–3500 and 1900–1400 cm$^{-1}$) and the $CO_2$ band near 2360 cm$^{-1}$.

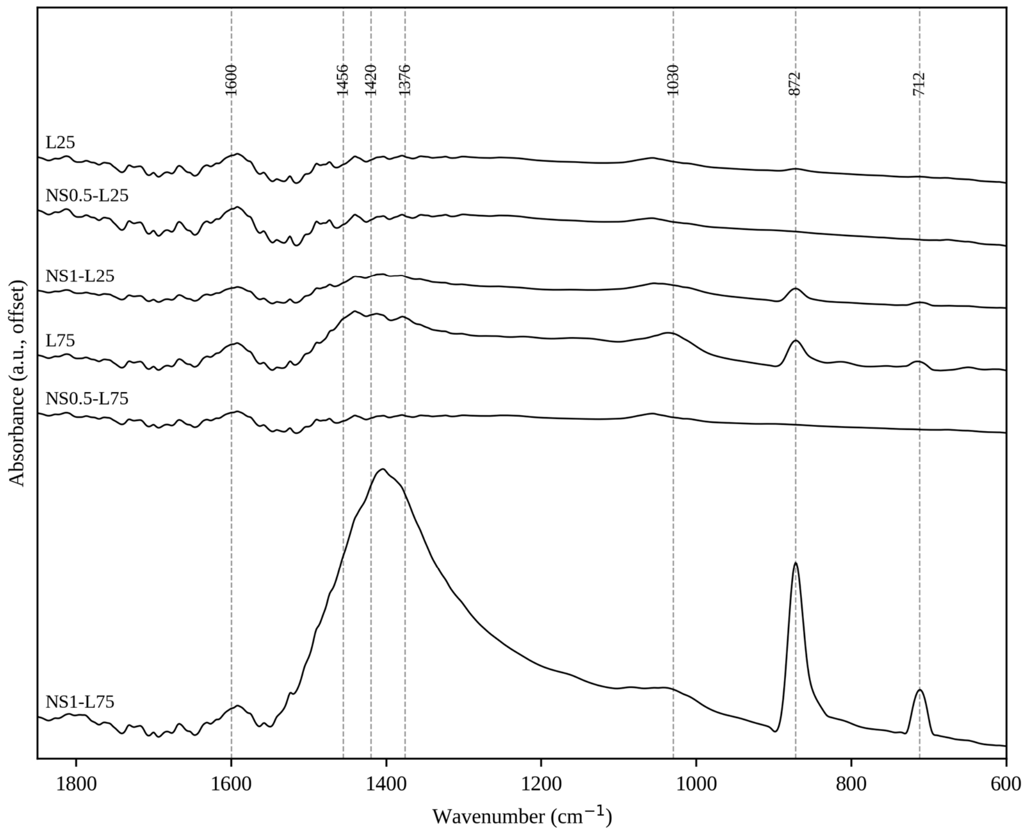


**Figure S2.** Spectra of the mixture fragments in the 1850–600 cm$^{-1}$ region, converted to absorbance (−log T), shifted to a common minimum and vertically offset for clarity (NS1-L75 with a larger offset because of its stronger carbonate bands). Dashed lines mark the band positions discussed in the article.

**Table S3.** Band positions (cm$^{-1}$) observed in the FTIR spectra. Reference positions and assignments follow references [42–46] and [24,31] of the article.

| **Assignment** | **Ref. position (cm$^{-1}$)** | **Bitumen** | **Kraft lignin** | **Nano-silica** | **L25** | **NS0.5-L25** | **NS1-L25** | **L75** | **NS0.5-L75** | **NS1-L75** |
|---|---|---|---|---|---|---|---|---|---|---|
| O–H stretching | 3350–3400 | — | 3350 | — | — | — | — | — | — | — |
| Aliphatic C–H asym. stretching | ~2920 | 2919 | 2939 | — | 2916 $^{w}$ | — | 2920 | 2919 | — | 2920 |
| Aliphatic C–H sym. stretching | ~2850 | 2852 | — | — | — | — | — | 2852 | — | 2853 |
| Aromatic C=C / skeletal | ~1600 | 1599 | 1599 | — | n.r. | n.r. | n.r. | n.r. | n.r. | n.r. |
| Aromatic skeletal (lignin) | ~1510 | — | 1507 | — | n.r. | n.r. | n.r. | n.r. | n.r. | n.r. |
| $CH_2$/$CH_3$ bending | ~1456 | 1456 | 1456 | — | n.r. | n.r. | n.r. | n.r. | n.r. | n.r. |
| $CO_3^{2-}$ asym. stretching (calcite), broad | ~1420 | — | 1420 $^{a}$ | — | — | — | ~1400 | ~1436 | — | ~1402 |
| $CH_3$ bending | ~1376 | 1373 | — | — | n.r. | n.r. | n.r. | n.r. | n.r. | n.r. |
| C–H in-plane deformation (guaiacyl) | ~1140 | — | 1138 | — | — | — | — | — | — | — |
| Si–O–Si asym. stretching | 1080–1100 | — | — | 1079 | — | — | — | — | — | — |
| S=O (bitumen) / C–O (lignin) | 1030–1060 | 1029 | 1032 | — | 1056 $^{w}$ | 1058 $^{w}$ | 1055 $^{w}$ | 1036 | 1056 $^{w}$ | — |
| Si–OH | ~950 | — | — | 959 | — | — | — | — | — | — |
| $CO_3^{2-}$ out-of-plane bending (calcite) | ~872 | — | — | — | — | — | 872 | 872 | — | 872 |
| Aromatic C–H out-of-plane bending | ~865, ~810 | 868, 808 | — | — | — | — | — | — | — | — |
| Si–O–Si sym. stretching | ~800 | — | — | 799 | — | — | — | — | — | — |
| $(CH_2)_n$ rocking | ~725 | 725 | — | — | — | — | — | — | — | — |
| $CO_3^{2-}$ in-plane bending (calcite) | ~712 | — | — | — | — | — | 711 | 715 | — | 711 |
| Si–O–Si bending | ~460 | — | — | 460 | — | — | — | — | — | — |

Positions are local absorbance maxima; for the broad carbonate band of the mixtures, the maximum of the smoothed spectrum (Savitzky–Golay, 41 points) is given. —: not detected. n.r.: not resolved, because the region overlaps the atmospheric water-vapour bands in the weak mixture spectra. [w] Weak band, close to the noise level. [a] In lignin, the band near 1420 $cm^{-1}$ arises from aromatic skeletal vibration combined with C–H in-plane deformation, not from carbonate.

## Section S3. Scanning electron microscopy

**Table S4.** SEM imaging conditions.

| Parameter | Value |
|---|---|
| Microscope | ZEISS Sigma 300 VP field-emission SEM (Carl Zeiss Microscopy GmbH, Oberkochen, Germany) |
| Detector/vacuum mode | Secondary electrons (SE2) / high vacuum |
| Accelerating voltage | 20 kV |
| Aperture | 30 µm |
| Specimen preparation | Fragments of fractured Marshall specimens, sputter-coated with gold |
| Working distance | 4.6–9.2 mm (L25 4.9–5.9; NS0.5-L25 5.4–8.7; NS1-L25 6.0–8.2; L75 8.6–9.2; NS0.5-L75 4.6–6.0; NS1-L75 8.3–8.9 mm) |
| Areas per specimen | Two |
| Magnification (pixel size) | 100× (1.12 µm), 300× (372 nm), 500× (223 nm), 1000× (112 nm), 3000× (37.2 nm), 5000× (22.3 nm), 10 000× (11.2 nm), 20 000× (5.6 nm) |
| Image size | 1024 × 768 pixels (instrument data bar removed in Figures S3–S8) |

Values were read from the metadata embedded in the TIFF files. For NS1-L75, no 20 000× image was recorded in area 1. Scale bars in Figures S3–S8 were redrawn from the pixel size of each image.

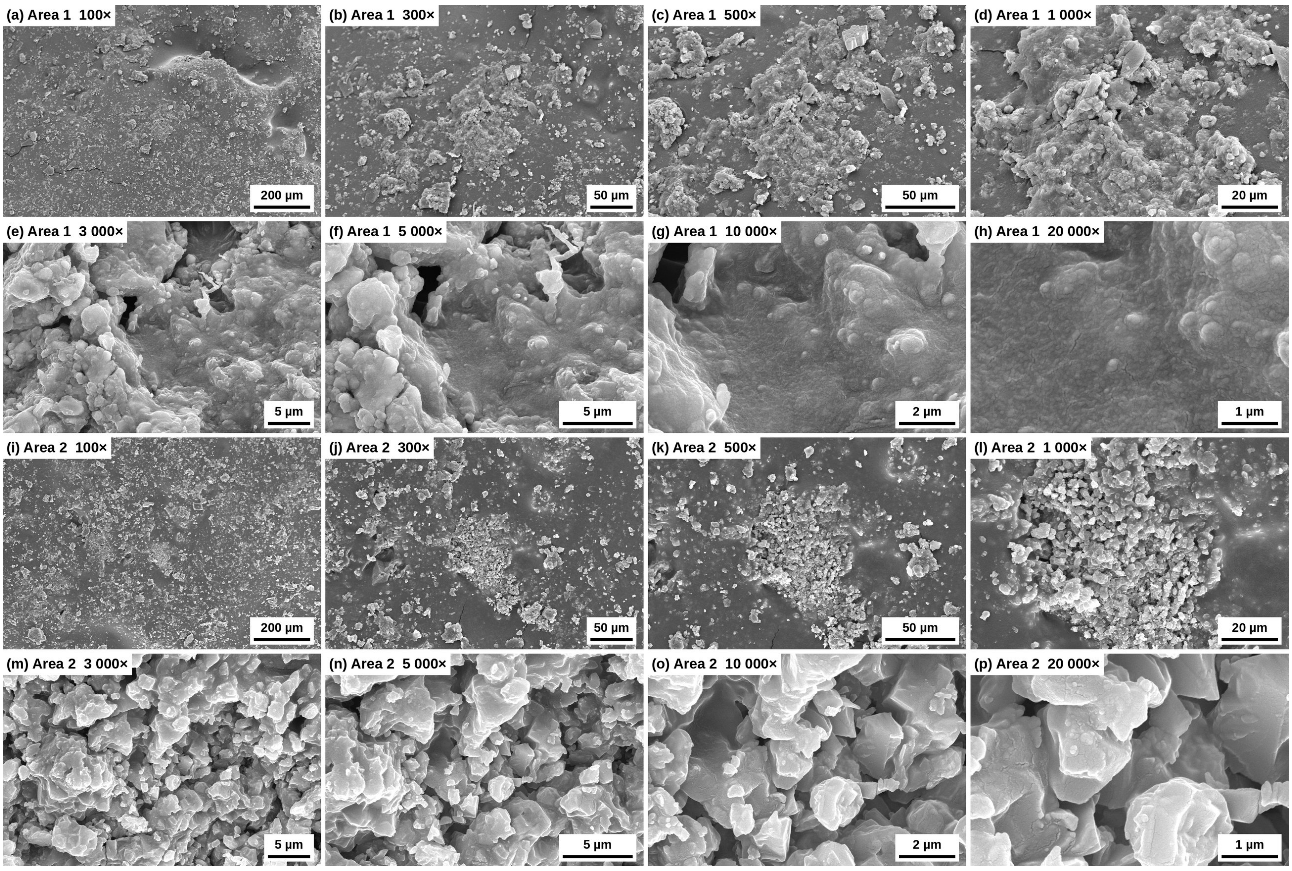


**Figure S3.** Complete SEM image series of a fracture-surface fragment of mixture L25: two areas, each imaged from 100× to 20 000× (SE2 detector, 20 kV, gold-coated). Panels (a–h) show area 1 and panels (i–p) area 2. Panels (d) and (f) are those shown in Figure 10 of the article.

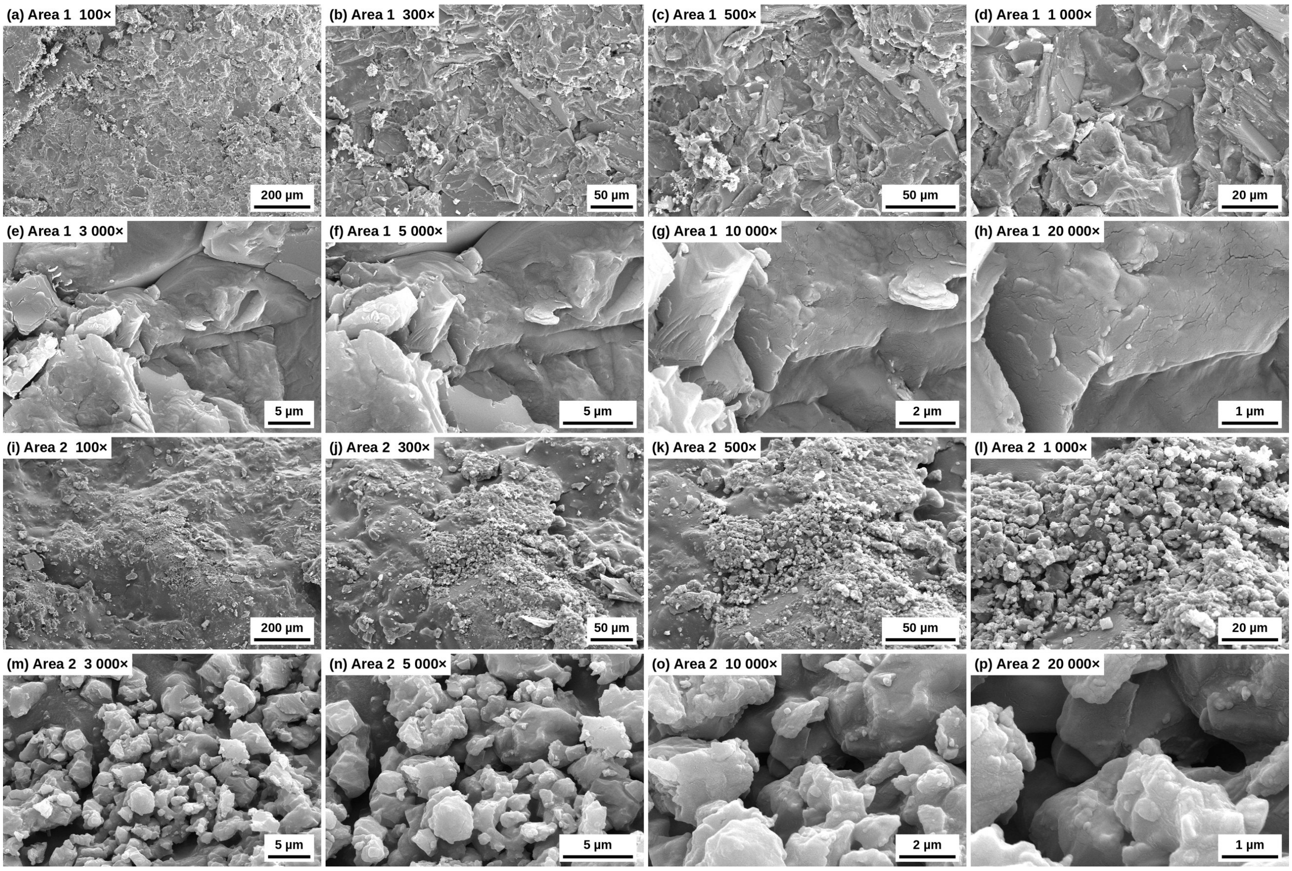


**Figure S4.** Complete SEM image series of a fracture-surface fragment of mixture NS0.5-L25: two areas, each imaged from 100× to 20 000× (SE2 detector, 20 kV, gold-coated). Panels (a–h) show area 1 and panels (i–p) area 2. Panels (d) and (f) are those shown in Figure 10 of the article.

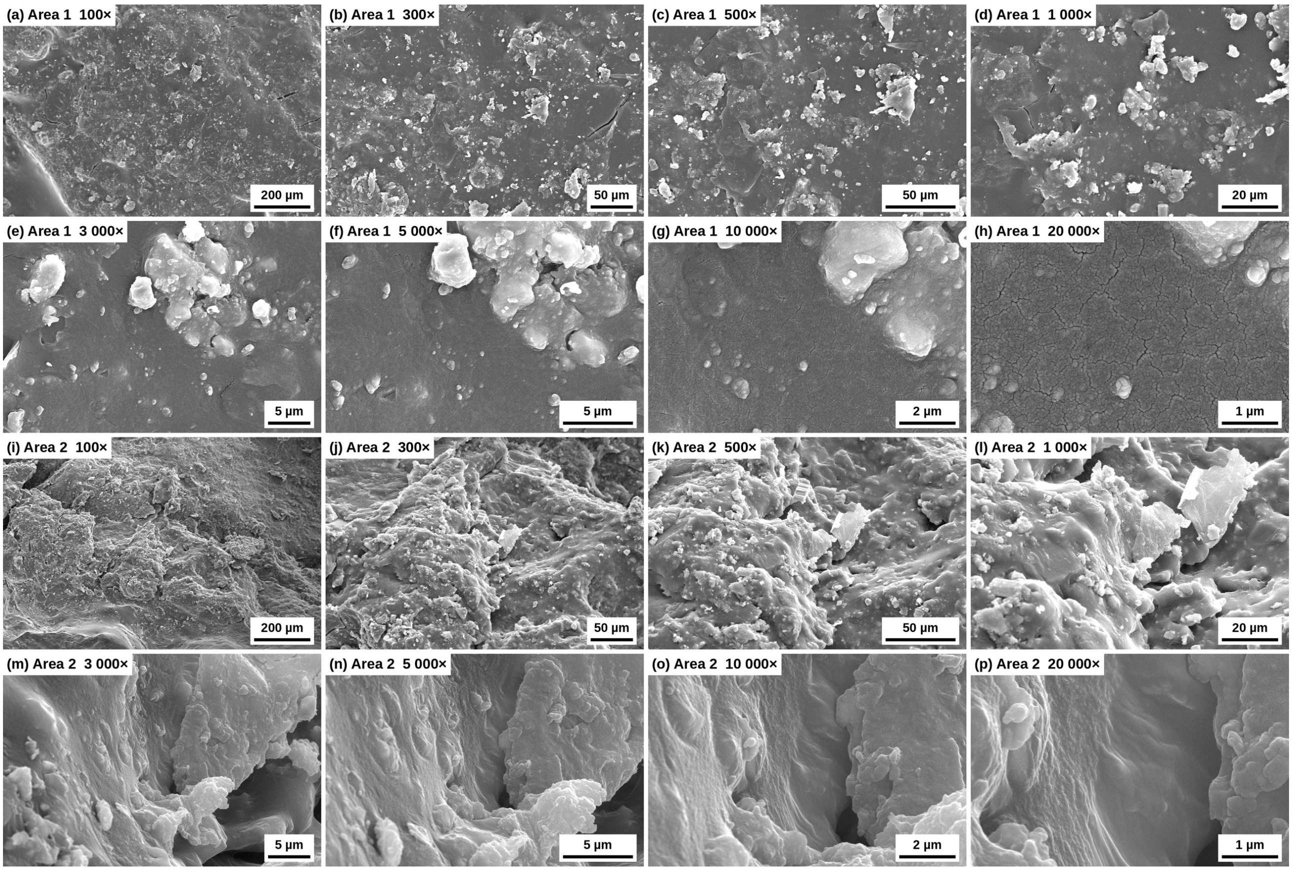


**Figure S5.** Complete SEM image series of a fracture-surface fragment of mixture NS1-L25: two areas, each imaged from 100× to 20 000× (SE2 detector, 20 kV, gold-coated). Panels (a–h) show area 1 and panels (i–p) area 2. Panels (d) and (f) are those shown in Figure 10 of the article.

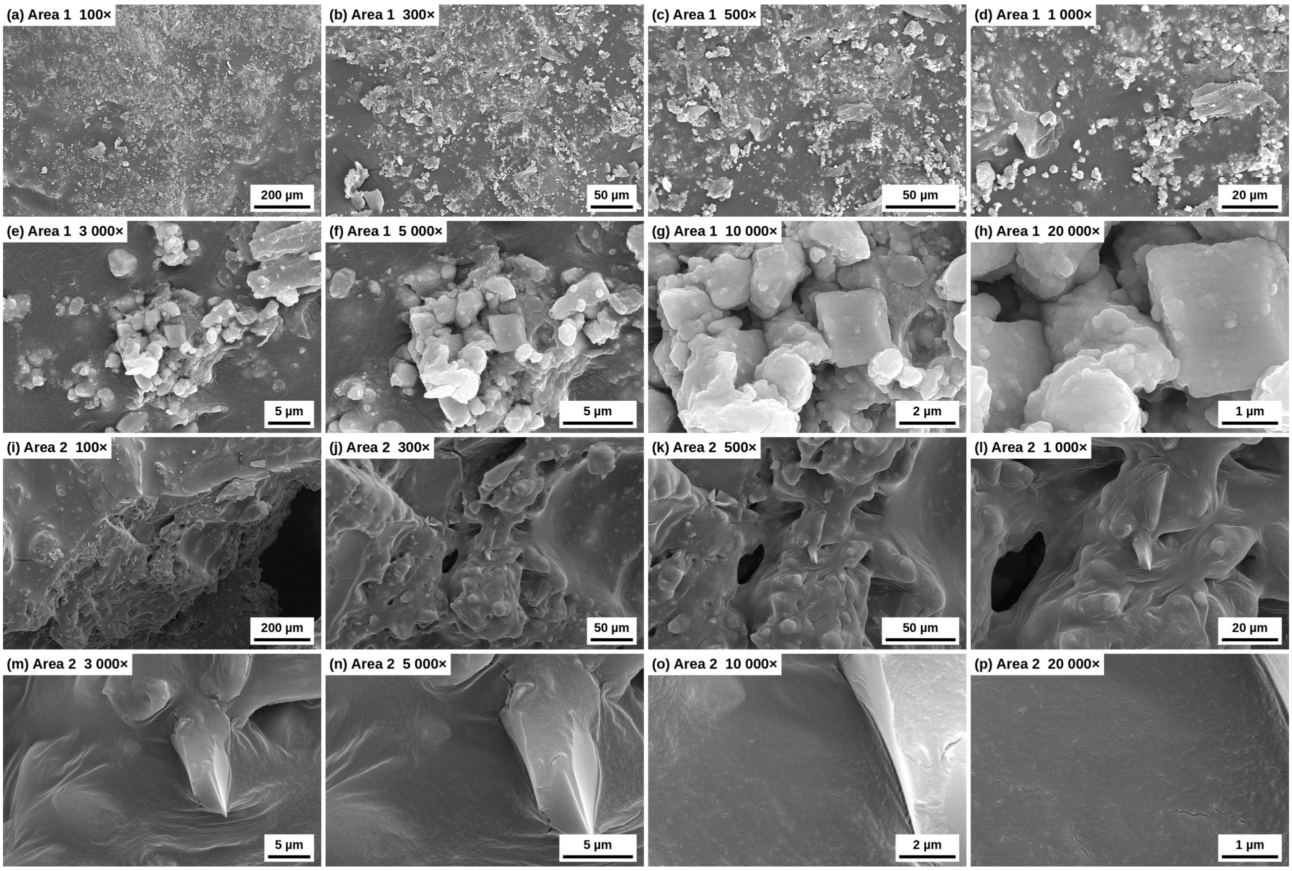


**Figure S6.** Complete SEM image series of a fracture-surface fragment of mixture L75: two areas, each imaged from 100× to 20 000× (SE2 detector, 20 kV, gold-coated). Panels (a–h) show area 1 and panels (i–p) area 2. Panels (d) and (f) are those shown in Figure 10 of the article.

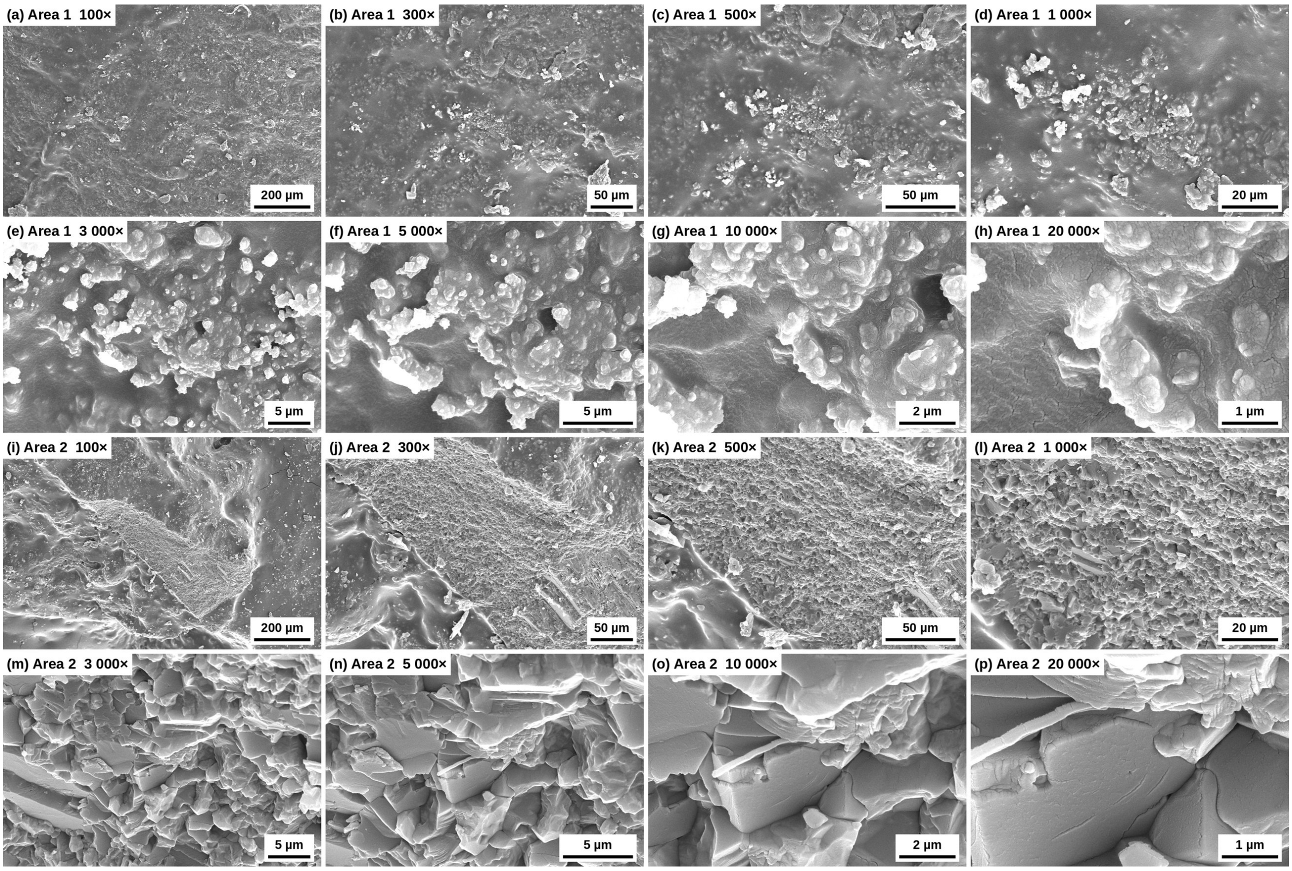


**Figure S7.** Complete SEM image series of a fracture-surface fragment of mixture NS0.5-L75: two areas, each imaged from 100× to 20 000× (SE2 detector, 20 kV, gold-coated). Panels (a–h) show area 1 and panels (i–p) area 2. Panels (d) and (f) are those shown in Figure 10 of the article.

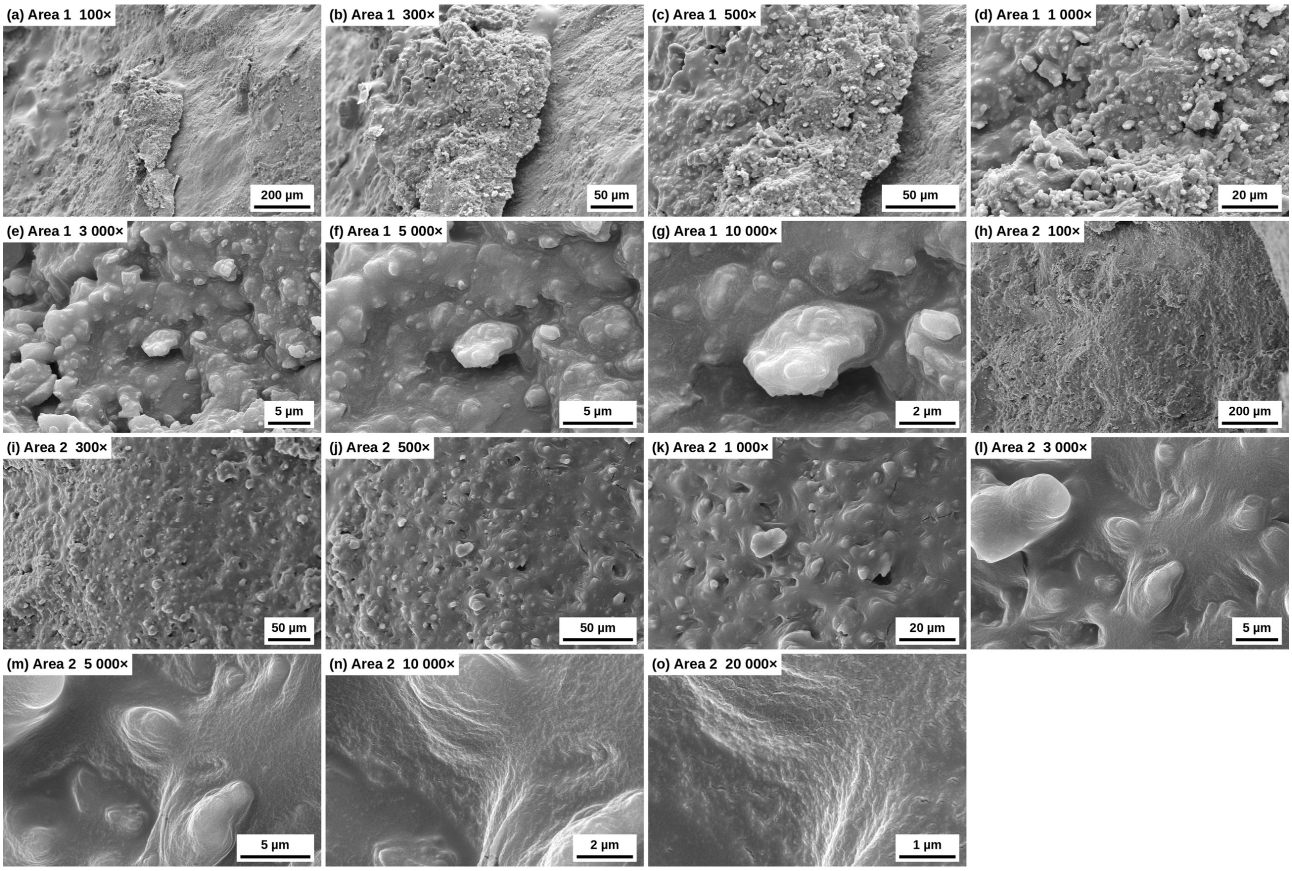


**Figure S8.** Complete SEM image series of a fracture-surface fragment of mixture NS1-L75: two areas, each imaged from 100× to 20 000× (SE2 detector, 20 kV, gold-coated). Panels (a–g) show area 1, for which no 20 000× image was recorded, and panels (h–o) area 2. Panels (d) and (f) are those shown in Figure 10 of the article.
